# Supplementary material for: Molecular organization of fibroin heavy chain and mechanism of fibre formation in Bombyx mori
Source: Commun Biol. 2024 Jun 29;7:786. doi: 10.1038/s42003-024-06474-1 (PMC11217467; doi:10.1038/s42003-024-06474-1)
Supplement: Supplementary file 2 — Supplementary Information [file 42003_2024_6474_MOESM2_ESM.pdf]

Supplementary Information for

Molecular organization of fibroin heavy chain and mechanism of fibre formation in *Bombyx mori*.

R.O. Moreno-Tortolero,[1,2*] Y. Luo,[3] F. Parmeggiani,[1,3] N. Skaer,[4] R. Walker,[4] L. Serpell,[5] C. Holland,[6] S.A. Davis[1*]

Corresponding author: ro.morenotortolero@bristol.ac.uk, s.a.davis@bristol.ac.uk

Supplementary Note

1- NOESY

Within the entire sequence of FibH, Y is only found next to A a total of 13 times, out of the 275 Y residues present. However, the ^{1}H- ^{1}H NOESY experiments showed consistent NOE signals between the A βH and Y δ,εH (aromatic). It is known that NOE signal depends inversely on the interatomic distance to the sixth power; or alternatively, the distance is directly proportional to the intensity to the -1/6 power. Thus, generally for NOE signal to appear, the two atoms need to be within about 6 Å. In fact, after using the internal Y interatomic distances as mean of calibration, it was possible to estimate average distances between A βH and Y δH to be about 3.4 Å and between A and Y εH 3.3 Å. Although these might be underestimated. There are 221 Y residues within the repetitive domains in total, with an estimated of 74 motifs that would place Y next to A in an inter-strand motif as described in the main text (Figure 2), thus after this adjustment, the estimated distance would be about 2.2-2.3 Å between the aromatic protons of Y and A βH, in an almost perfect agreement with the model (ca. 2.5 Å). Similar calculations can be made for the observed and expected NOEs from Y to S or V, where Y to S are expected to be similarly across strand distances at least 19 times, and Y to V intra-strand distances (motifs VGY) a total of 27 times, the expected distances are of about 2.4-2.8 Å and about 3.2-3.7 Å, while the corrected measured distances are 2.1 Å and 2.3 Å, respectively.

2- Polymer viscosity

For polymers beyond a critical entanglement concentration, the viscosity follows a power law proportionality to the molecular weight as shown in Equation 1.

$$\eta = kM^{a} \qquad \text{Equation 1}$$

Where η is the shear viscosity, k is a polymer dependent constant, M is the molecular weight, and a is the proportionality exponent, which for most polymers assumes the value of 3.5 with very low deviation for a myriad of polymers. So much so that the proportionality is often directly expressed with this value. Consequently, $\eta \propto M^{3.5}$ and thus, if one considers the relative molecular weight reduction observed in the SDS-PAGE, it is about half of the original MW. This reduction in MW would be translated in a proportional reduction of η by $(0.5)^{3.5}$, or 0.088.

Furthermore, viscometry analysis of the samples, NLSF and RSF, was conducted in the LiBr solutions at 20 wt% of concentration. The standing assumption is that in the LiBr solution, the protein is in its statistical coil conformation as a polymer in solution. As it would be expected, under these conditions, the value of the zero shear viscosity (η_0) for NLSF and RSF are 58.1 ± 0.2 and 2.13 ± 0.02 Pa.s, respectively. One order of magnitude difference was predicted from the previous power law relationship. The fitting of the experimental curves was done using the Cross method (see equation contained within Figure S19), where viscosity as a function of shear rate ($\dot{\gamma}$), is fitted using the infinite shear viscosity (η_∞), η_0, a relaxation time (τ) and a power coefficient (m).

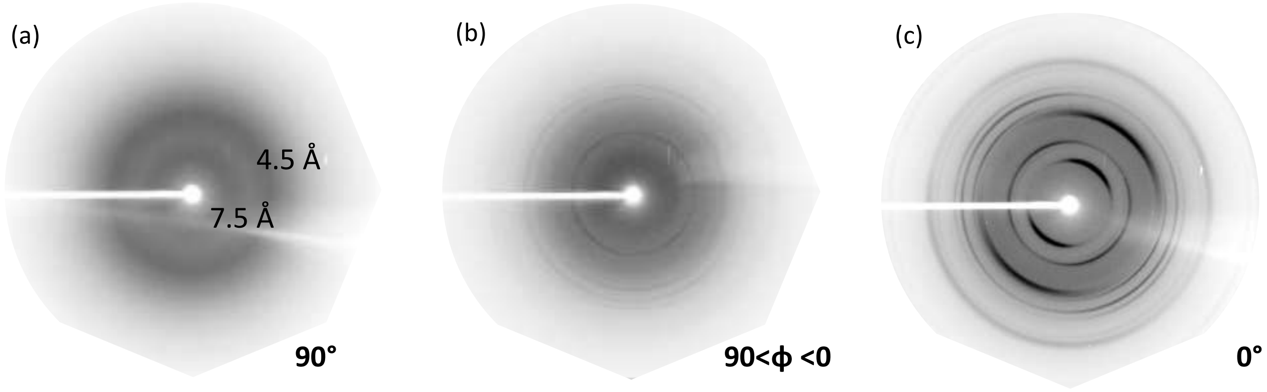

Figure S1.

2D X-ray diffraction in Fibre diffraction obtained from Silk-I film obtained from NLSF at different illumination angles relative to the film plane. Diffraction pattern obtained at 0° (a), between 0 and 90° (b) and 90° illumination (c).

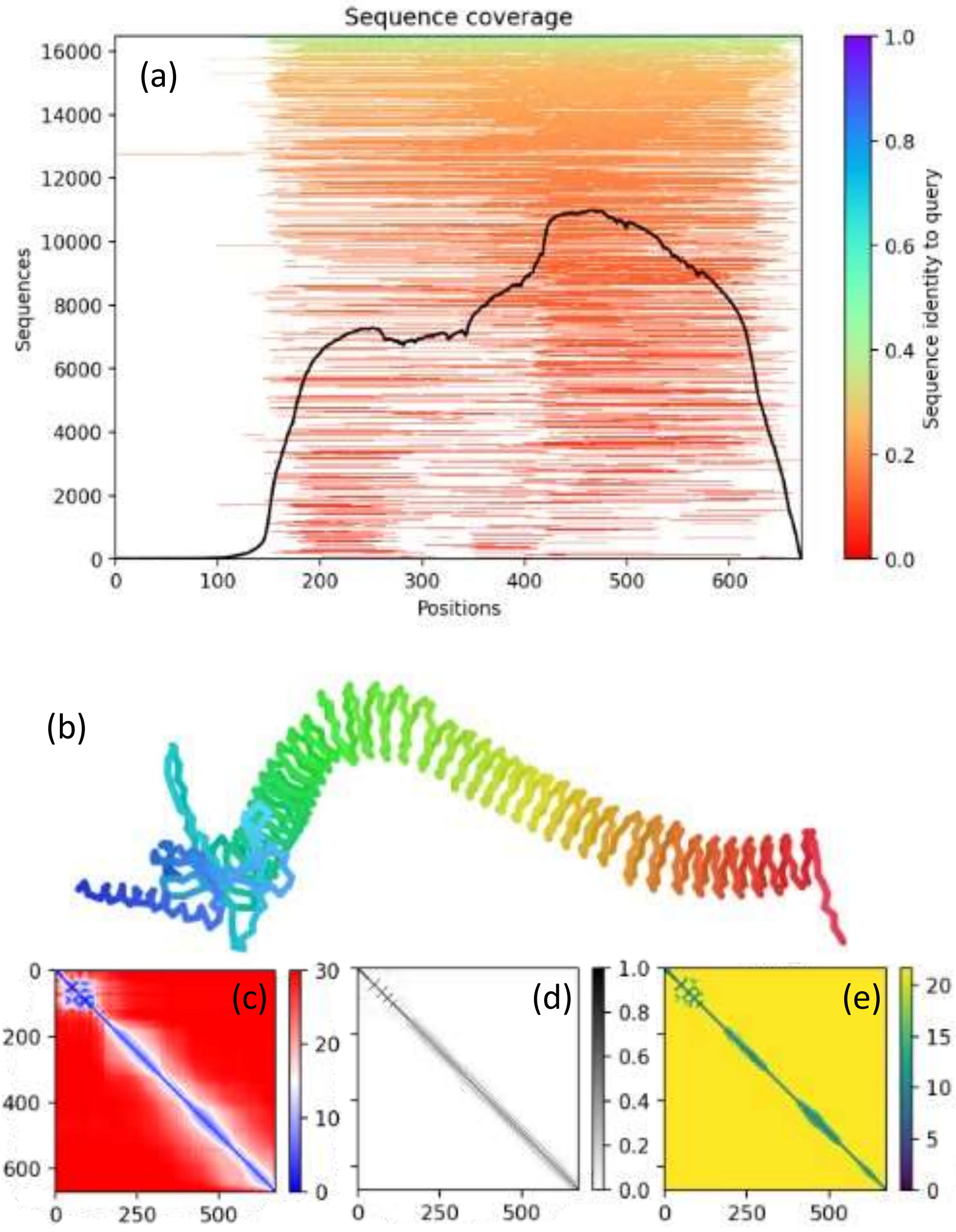

Figure S2.

AF2 simulation of NTD and first repetive domain. (a) Multisequence alignment (MSA) results from a jackhammer database search showing +16000 found sequences aligned to the sequence of the first 650 residues of FibH (NTD+ first repetitive domain). (b) Ribbon representation of one of the five obtained models showing β-solenoid conformation coloured blue to red in an N to C direction. 2D plots of the predicted alignment error (PAE, c), predicted contacts (PC, d) and predicted distogram (PD, e) for the shown model. In the 2D plots, sequence residue position is plotted both on the horizontal and vertical axis, and the predicted value is in the out-of-plane axis, with the range and coloured coding being shown on the right-hand side of each plot.

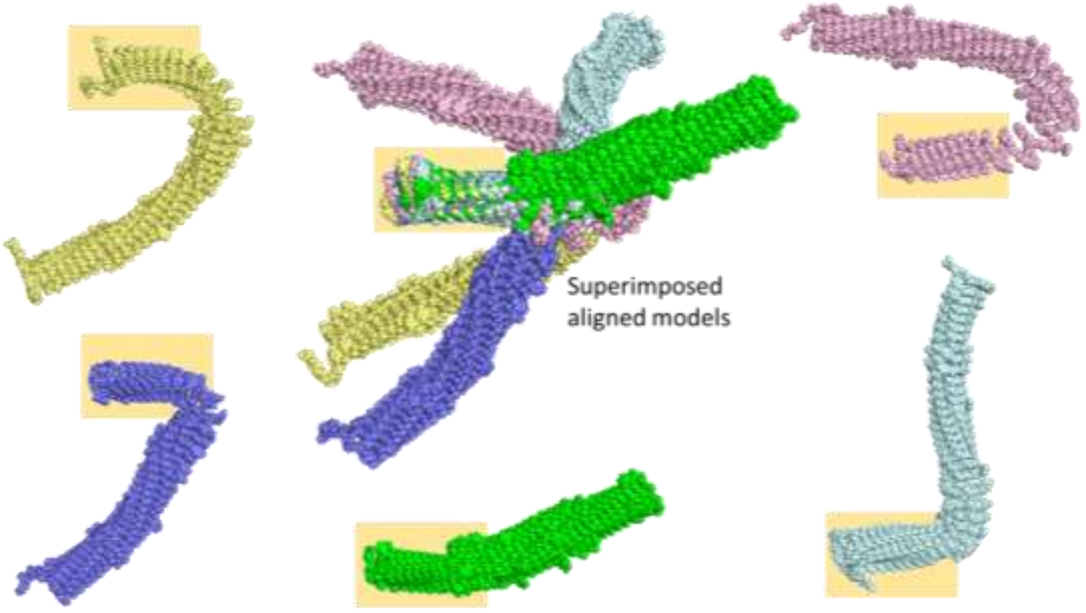

Figure S3.

Conservation of the β-solenoid topology for different generated models. Schematic representation of the five obtained models for the first repetitive domain, aligned at the apparent first rigid sub-domain and superimposed (centre) to illustrate possible flexibility of the solenoidal structure.

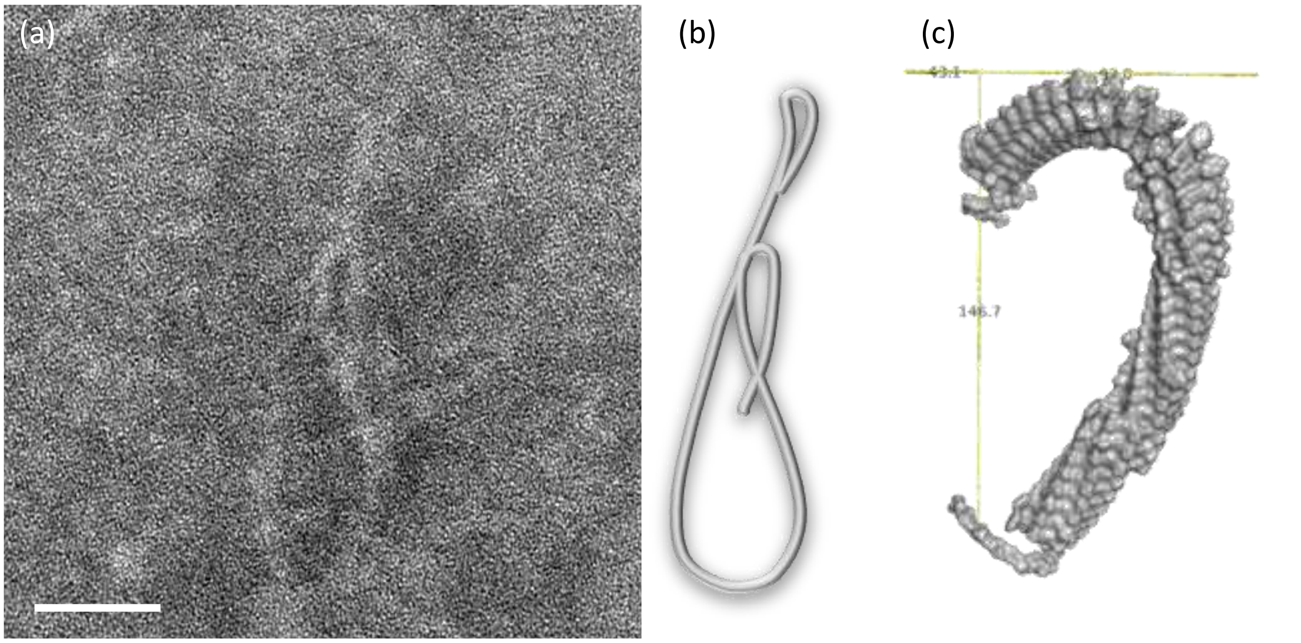

Figure S4.

Analysis and interpretation of TEM micrograph and FibH structure. TEM image obtained from lightly stained NLSF sample at pH 6 (a), a drawing showing interpretation of the observed profile (b) and a surface representation of a segment of the obtained model from the first repetitive domain showing similar curvature to that observed in the image (c). Scale bar 40 nm.

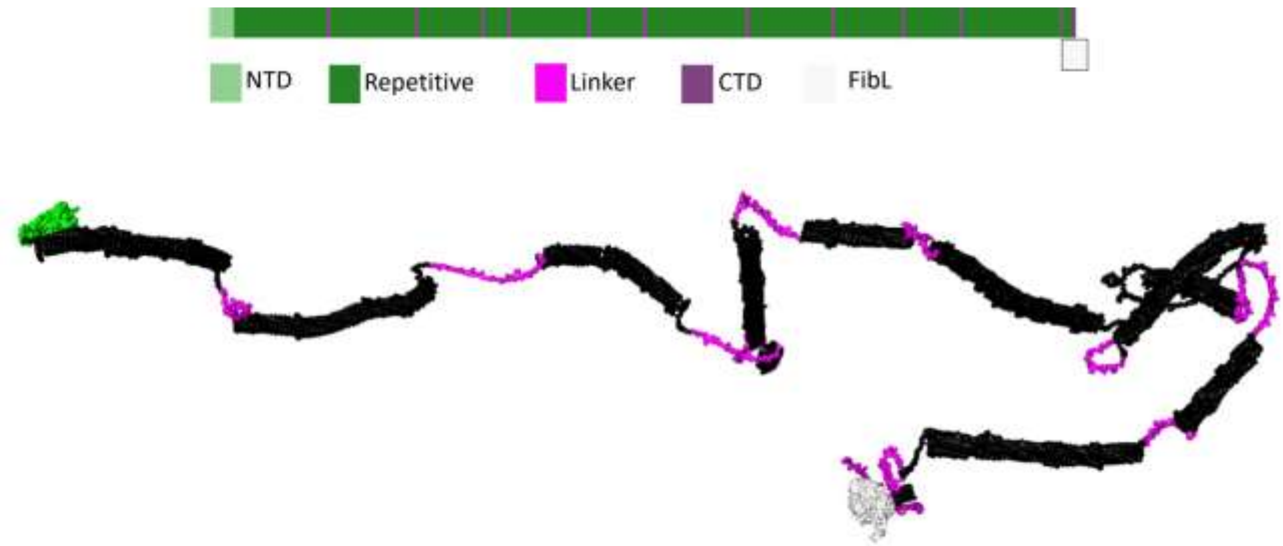

Figure S5.

Structure and topology of full FibH and FibL. At the top, a simplified cartoon of FibH plus FibL showing the multidomain structure at the primary level, with a legend of domains and colouring shown below. At the bottom is the proposed secondary level structure with the domains coloured following the previous cartoon.

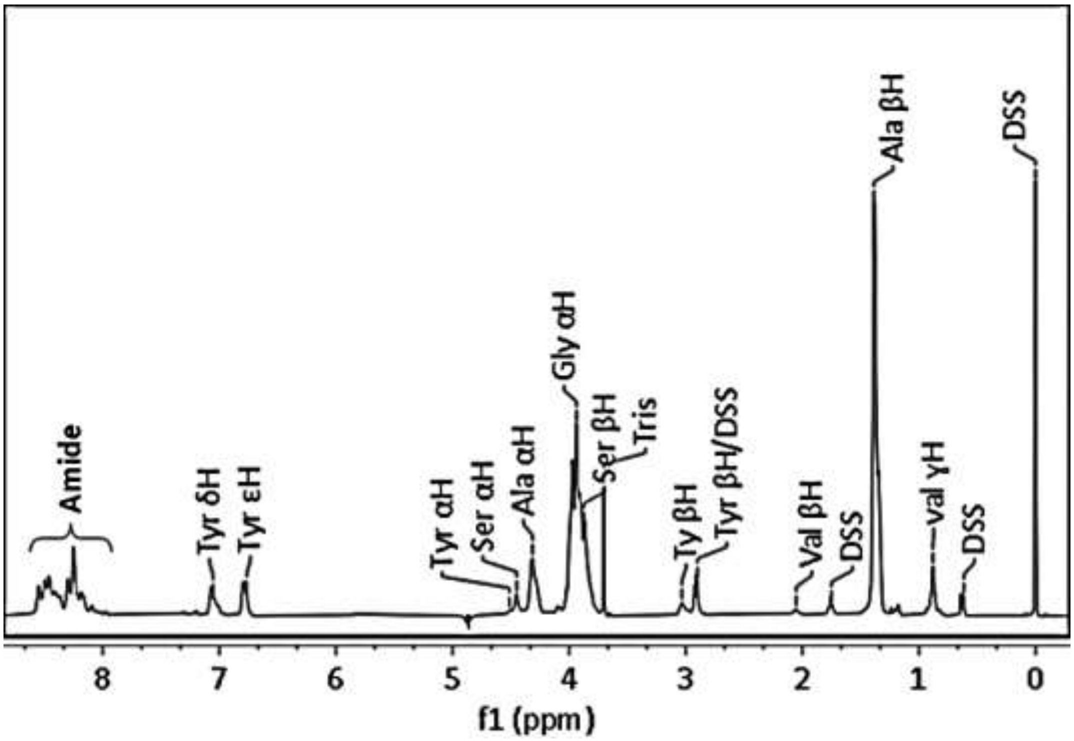

Figure S6.

One dimensional proton of fibroin. Simple proton spectra of partially deuterated NLSF with chemical shift assignment ran using a Bruker Advance III 700 MHz instrument at pH 6 and referenced with DSS.

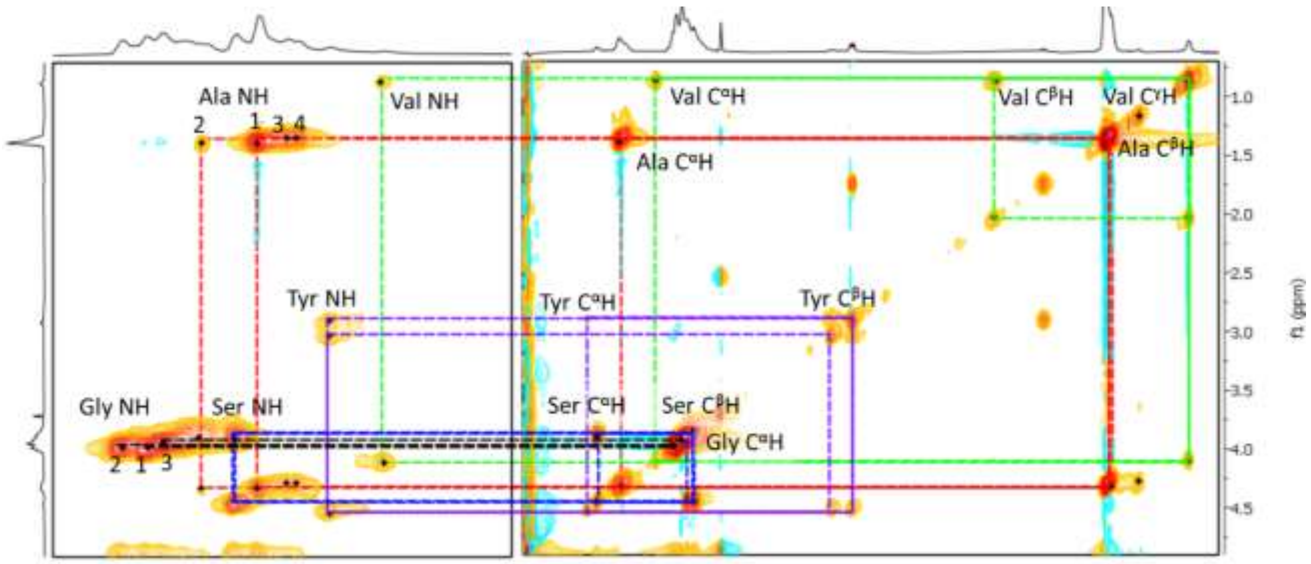

Figure S7.

Chemical shift assignment of proton. Homonuclear ^{1}H- ^{1}H TOCSY experiment conducted using a Bruker Advance III 700 MHz instrument at pH 6 and referenced with DSS of NLSF (5 mg/mL). Chemical shift assignment is shown regarding the amino acid and coupling connections shown in coloured boxes/lines. Boxes/lines are coloured by amino acid, with G, A, S, Y, and V is shown with black, red, blue, purple, and green lines, respectively).

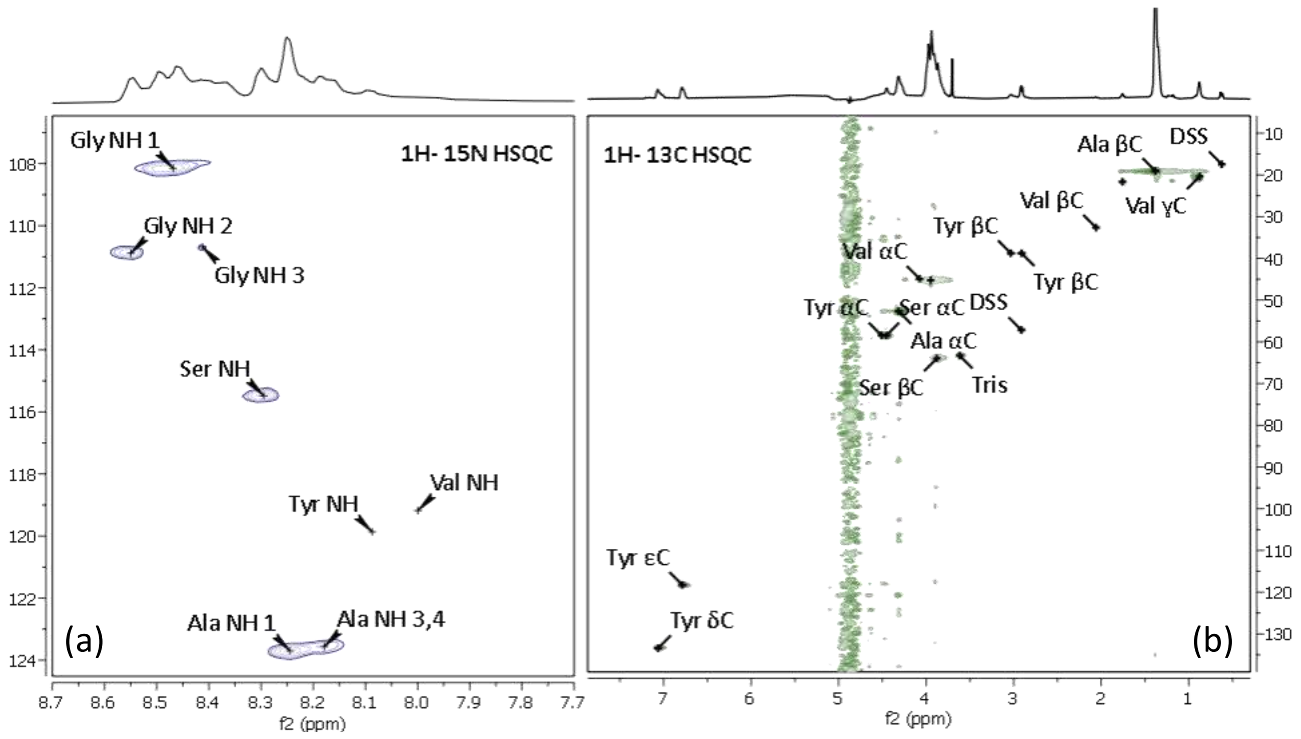

Figure S8.

NMR chemical shift residue assignment for NLSF. Heteronuclear ^{1}H- ^{15}N HSQC (a, blue) and ^{1}H- ^{13}C HSQC (b, green) with their respective shift assignment.

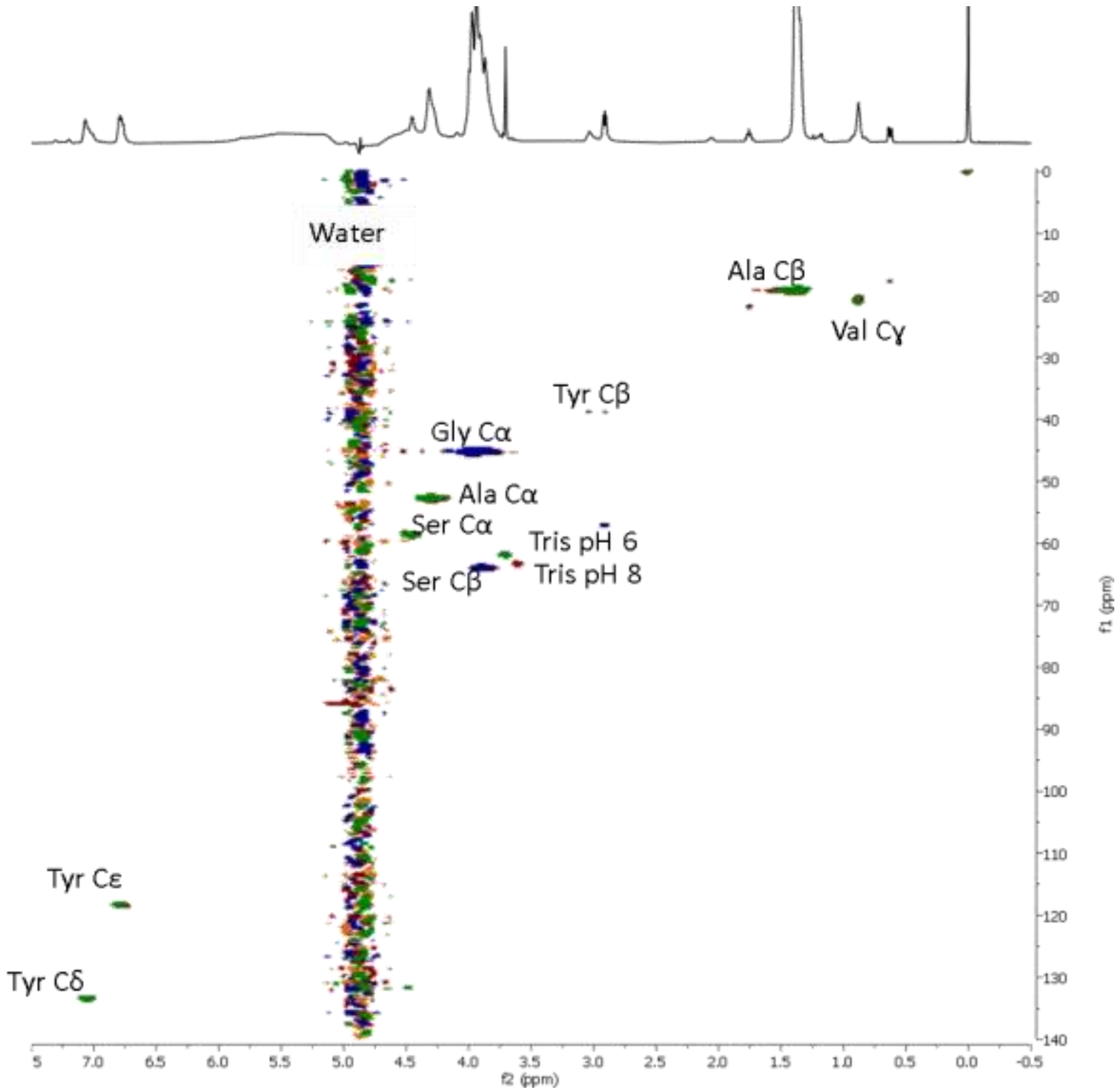

Figure S9.

Effect of pH on chemcial shifts of NLSF. Superimposed ^{1}H- ^{13}C HSQC spectra obtained for NLSF samples buffered at pH 6 and 8, showing no change in the chemical environment of the detectable residues.

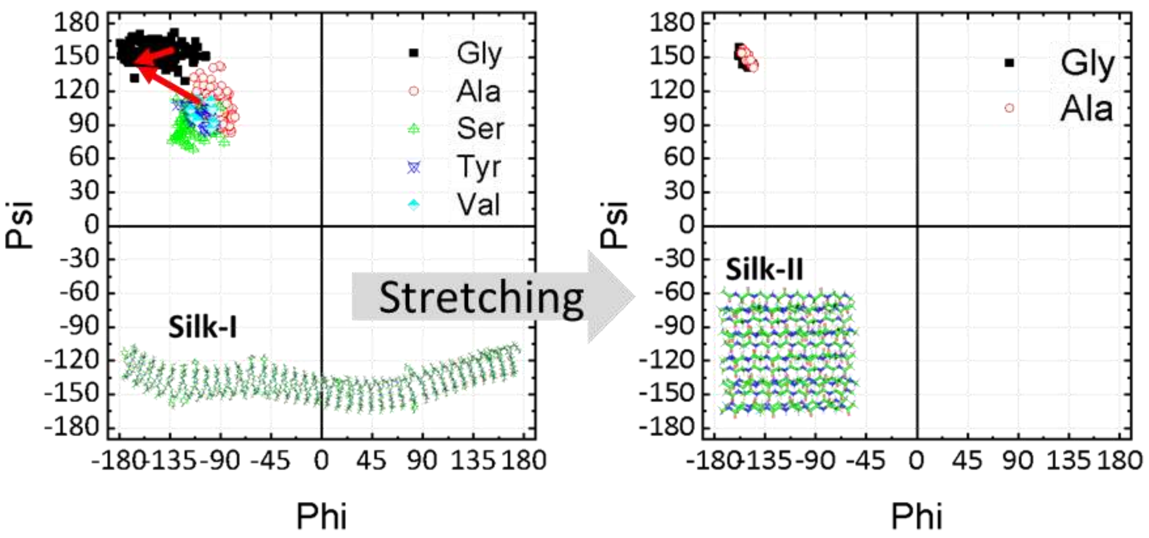

Figure S10.

Silk I to Silk II transformation, dihedral angles perspective. Proposed stretching induced transformation of the proposed Silk-I to the theoretical Silk-II (DOI: 10.5452/ma-cs24y) as observed from dihedral angles.

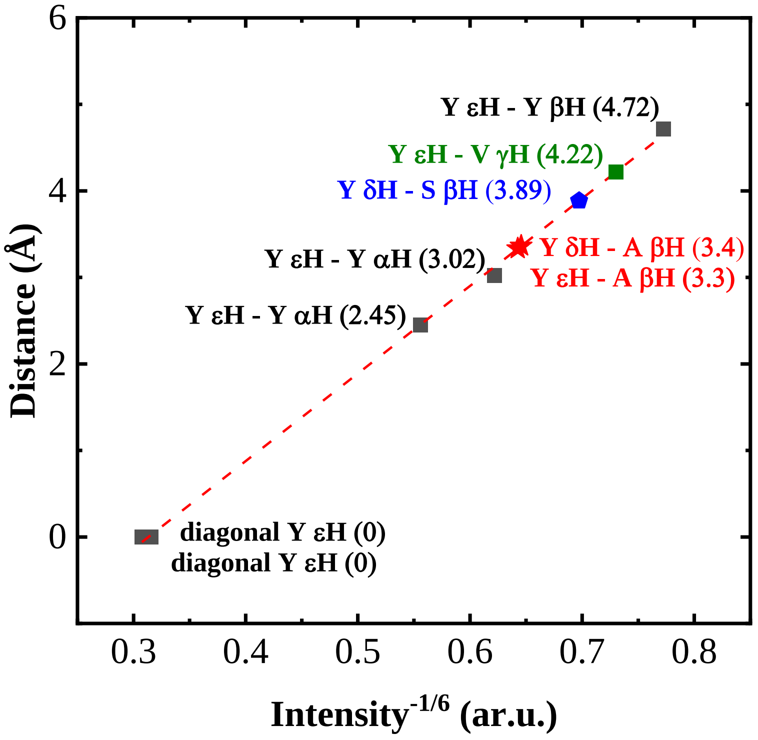

Figure S11.

Analysis and interpretation of NOESY data from NLSF. Distance against intensity $^{-1/6}$ for the assigned shifts and sticks representation of a Y residue showing estimated intra-residue distances used as internal calibration and estimated experimental values according to the internal calibration.

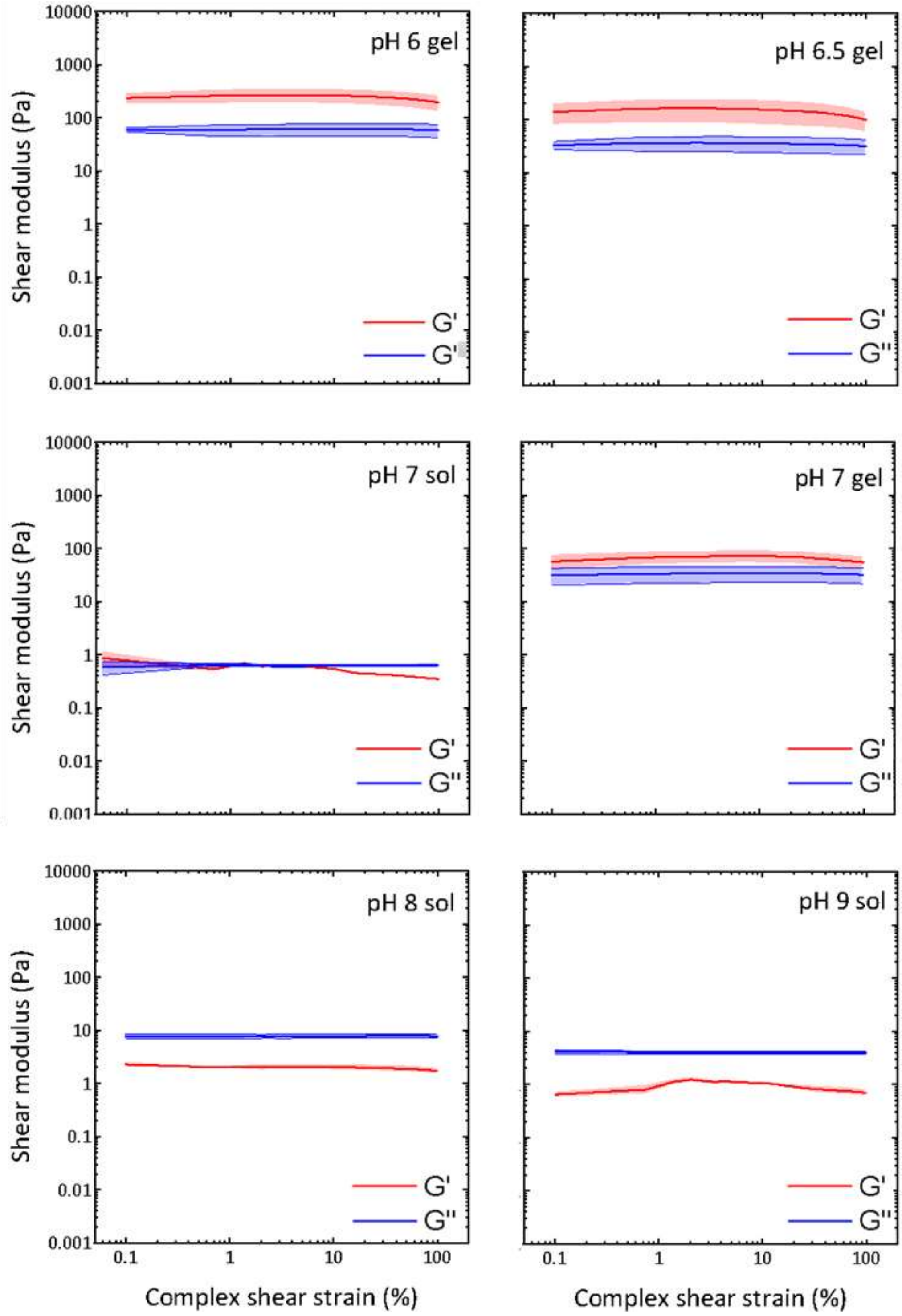

Figure S12.

Constant frquency rheology characterisation of NLSF. Shear rheology oscillatory experiment where the complex strain was varied between 0.1 and 100 % at a constant frequency (1 Hz) for the samples of NLSF buffered at different pH (indicated within the graphs). Curves are shown as averages of 5 replicates, with the standard deviation shown as the shadowed area around the solid line. Experiments were intended to determine the linear viscoelastic limit of the materials.

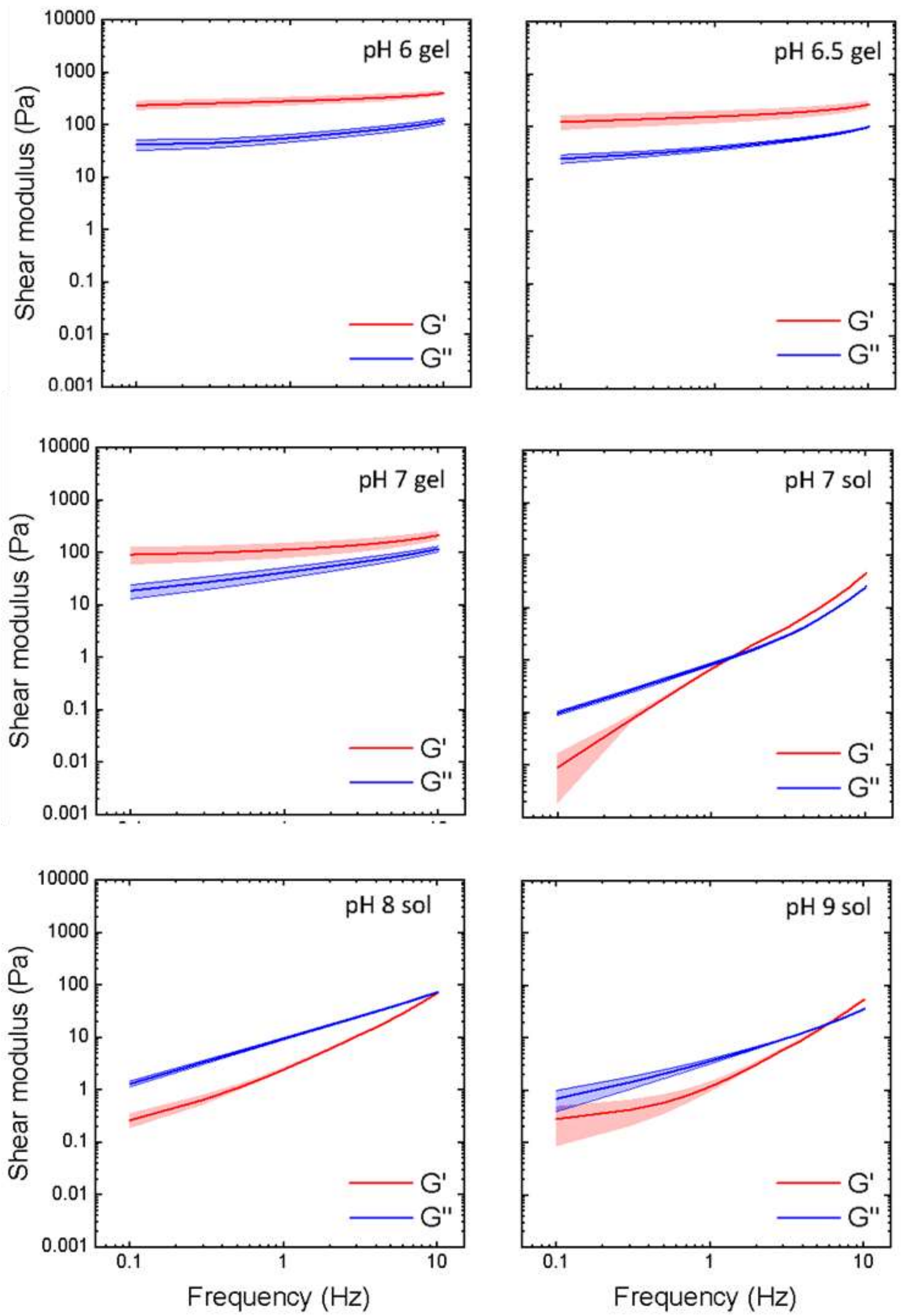

Figure S13.

Constant strain rheology characterisation of NLSF. Shear rheology oscillatory experiment where the frequency was varied between 0.1 and 10 Hz at a constant strain (2 %) for the samples of NLSF buffered at different pH (indicated within the graphs). Curves are shown as averages of 5 replicates, with the standard deviation shown as the shadowed area around the solid line. Experiment shows the clear sol-gel transition suffered by the material as the pH is lowered below 7.

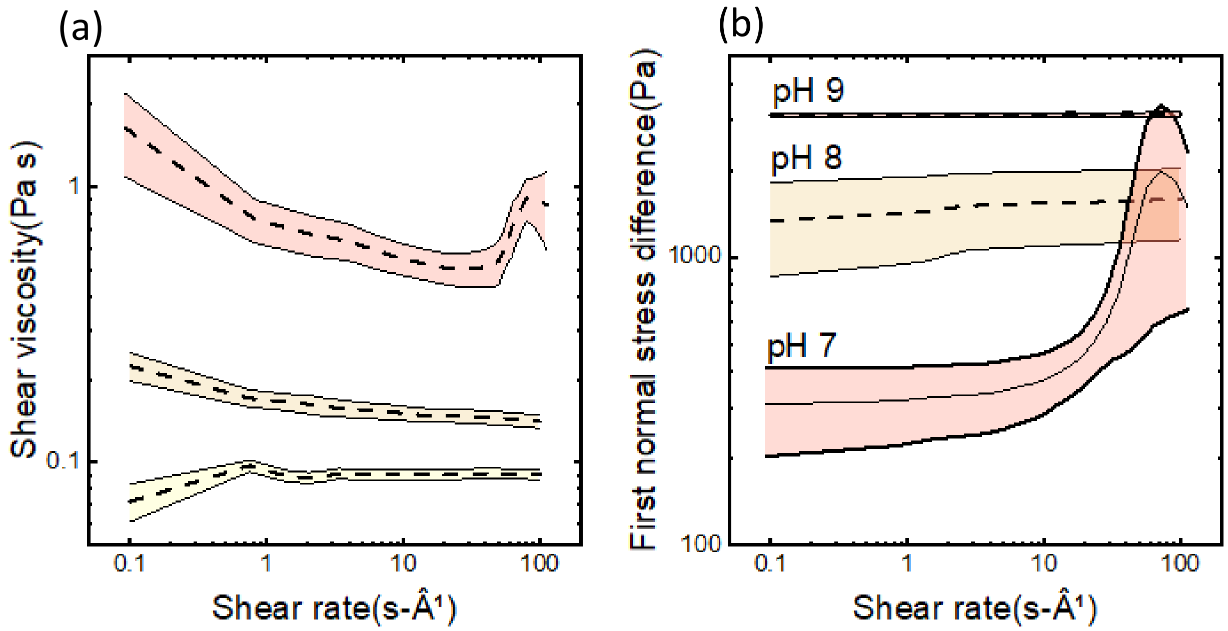

Figure S14.

Shear viscosity against shear rate for liquid samples of NLSF at different pH. Samples at pH (7, 8 and 9) and (a) and first normal force difference vs shear rate of same samples (b). Curves showed averages (dashed black lines) and standard deviation (shadowed area around average), technical replicates N = 5.

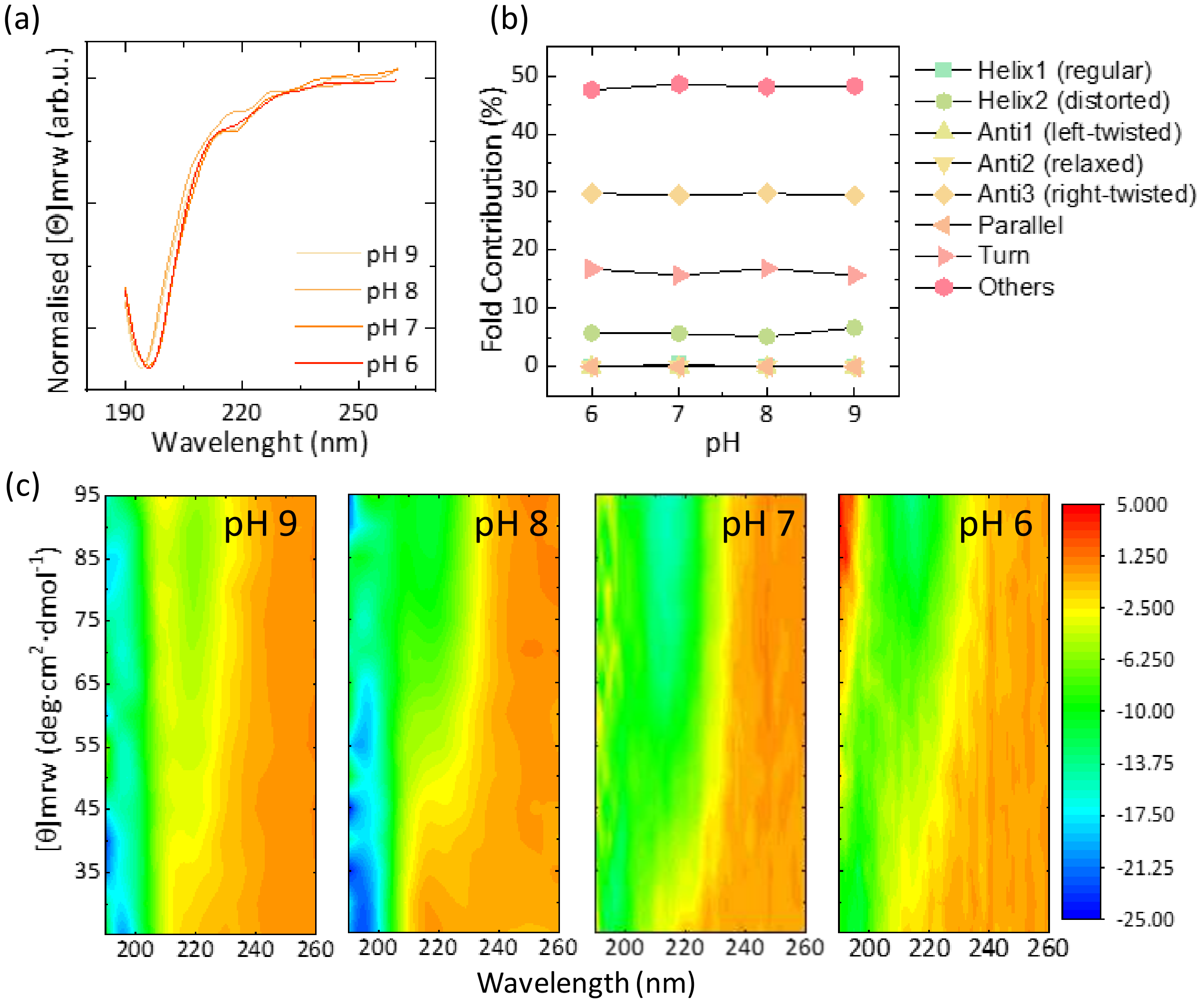

Figure S15.

Circular dichroism analysis of NLSF samples. Overlapped normalized Ellipticity values of the buffered samples obtained by far-UV CD spectroscopy (a). Bestsel deconvolution of the curves showing estimated structural contributions to the ellipticity signal (b). Series temperature scans of the buffered samples in the far-UV region, where the vertical axis is the temperature, horizontal axis the wavelength, and the far-right the out-of-plane scale with red being the maximum range and blue minimum (c).

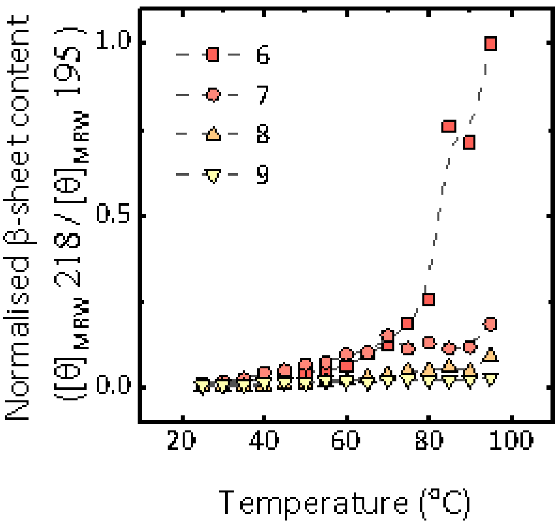

Figure S16.

Normalized β-sheet content as a function of temperature for all buffered samples (NLSF). The β-sheet content was estimated as the ratio between the ellipticity at 218 and 195 nm, normalized against the maximum obtained value for the data set.

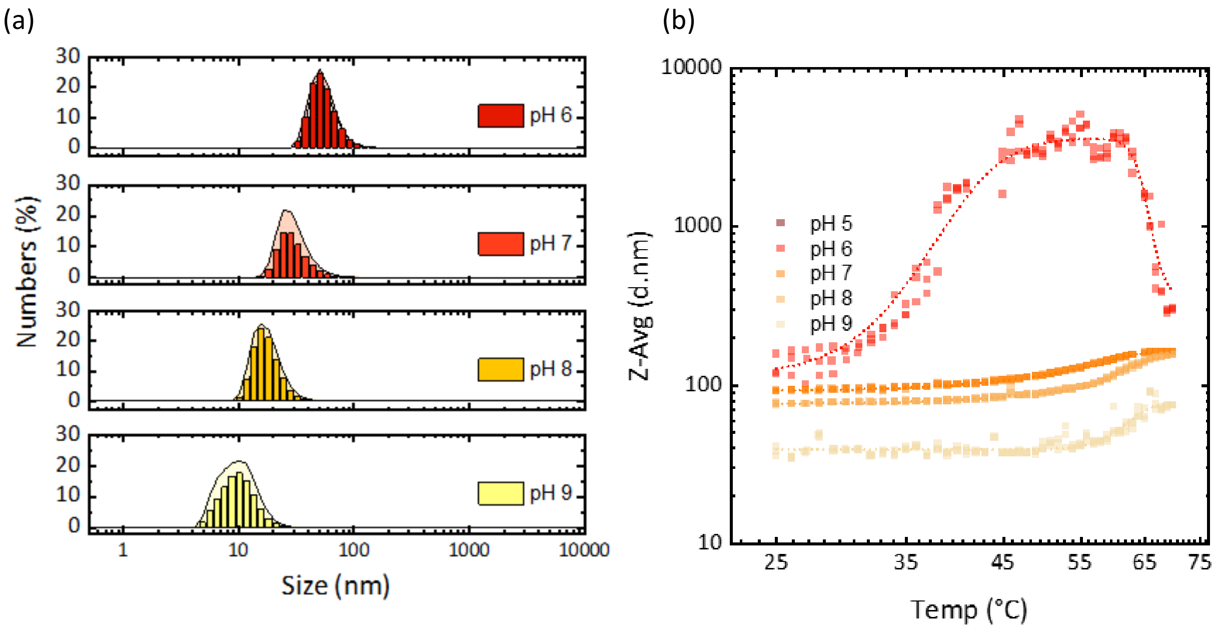

Figure S17.

Dynamic light scattering, DLS, analysis. DLS size distribution of NLSF obtained at different pH values plotted as the Numbers (%) against the size in a lineal vs log scale (a). Values of the Z-average size for the buffered NLSF samples as a function of temperature (b). Values presented for technical replicates (N: 3).

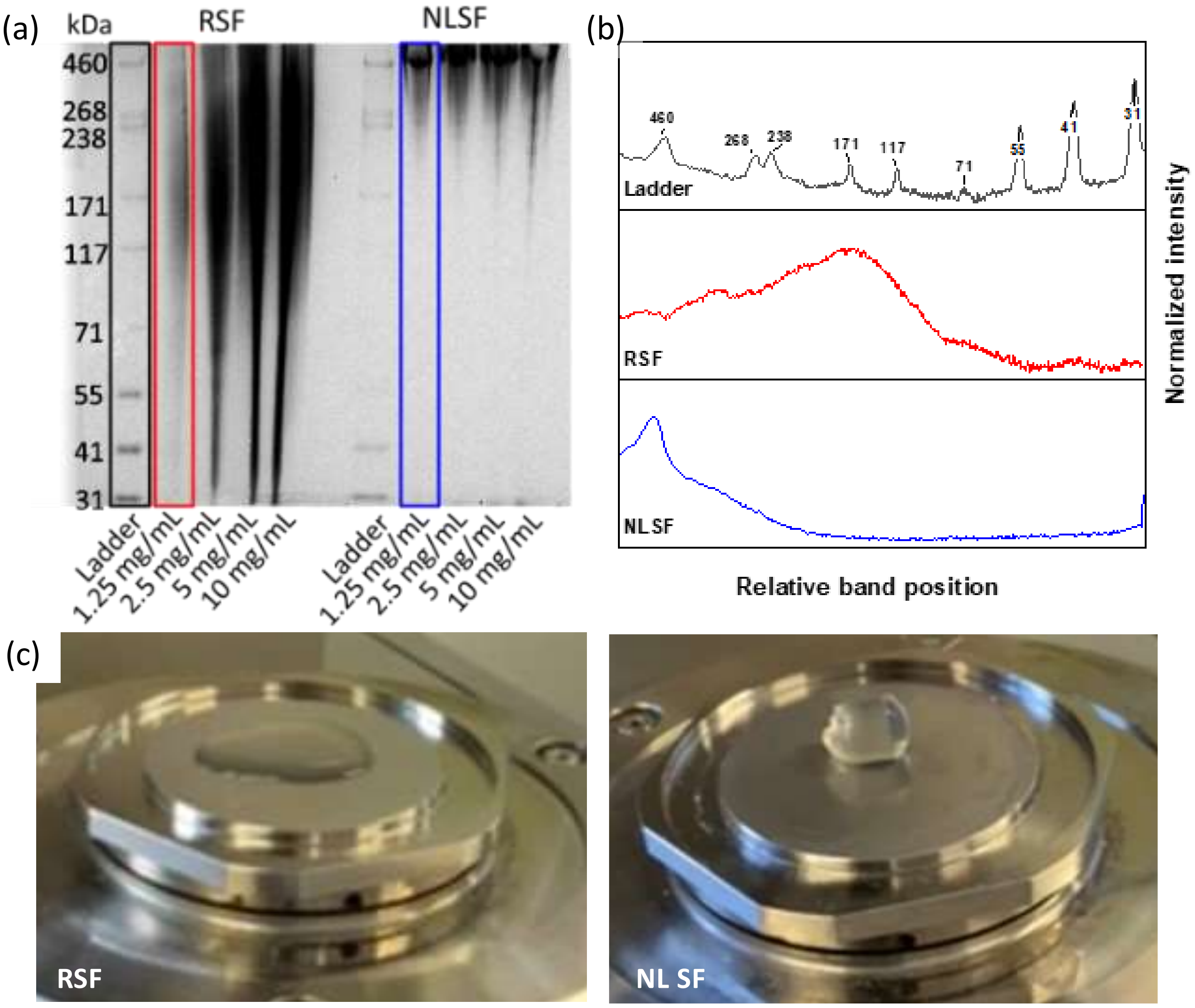

Figure S18.

Comparison of RSF and NLSF materials. Uncropped SDS-PAGE gel of RSF and NLSF at different protein concentrations with reference ladder next to them (a). profiles extracted from the gels image from the selected bands in rectangles (b). Photos of samples loaded onto a rheometer geometry of unbuffered RSF and NLSF indicated (c).

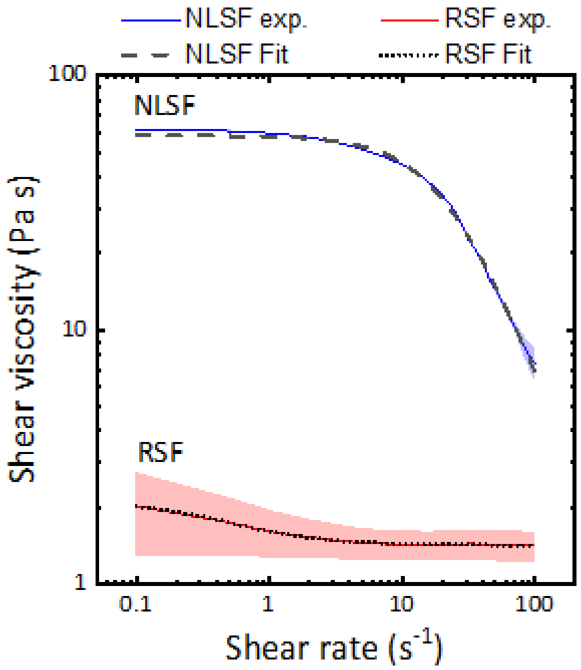

Cross fluid

$$\eta(\dot{\gamma}) \;=\; \eta_\infty + \frac{(\eta_0 - \eta_\infty)}{1 + (\tau\dot{\gamma})^m}$$

	NLSF	RSF
η_0	58.1 ± 0.2	2.13 ± 0.02
η_∞	0.9 ± 0.3	1.4255 ± 6E-4
τ	0.043 ± 3E-4	2.3 ± 0.1
m	1.48 ± 0.02	1.18 ± 0.03

Figure S19.

Viscometry analysis of silk ion LiBr solutions. Flow curves obtained for NLSF and RSF in their LiBr solutions plotted as shear viscosity against shear rate in a log-log scale. Solid lines represent averaged experimental data, and dotted or dashed lines represent fits to a Cross fluid model, with constitutive equation shown on the right, and the table below it shows the fitting parameters.

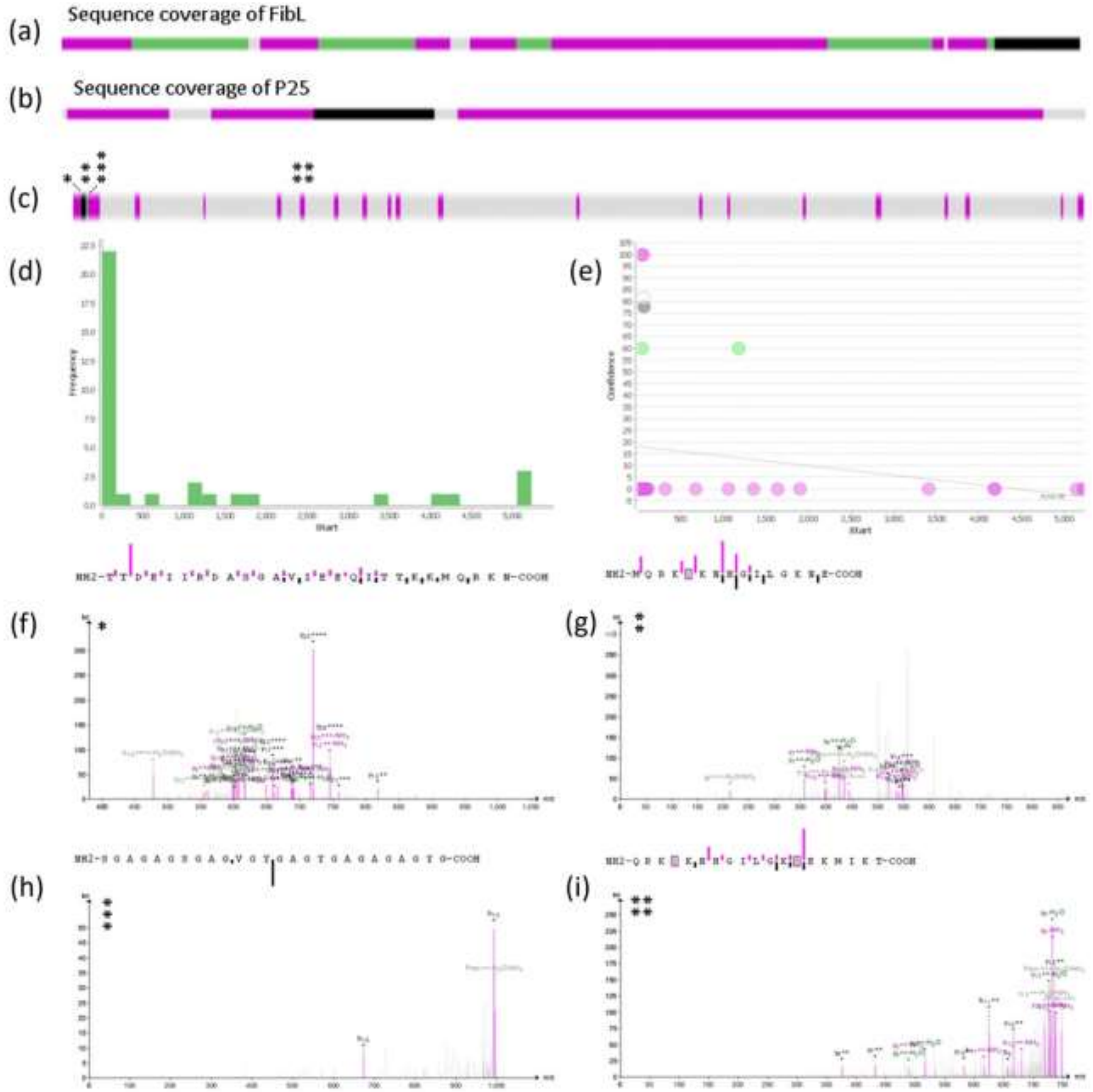

Figure S20.

Results from the proteomics analysis. Cartoons representing the sequence coverage of FibL (a) P25 (b)) and FibH (c). Plot representing the frequency distribution of peptides against residue position of the covered peptides from FibH (d). Plot representing the confidence of found peptides against their residue position (e). Series of example mass spectrograms obtained for the labelled peptides in FibH (f-i). Within the cartoons, the magenta section represents low confidence assignments, and green and black represent high confidence assignments.

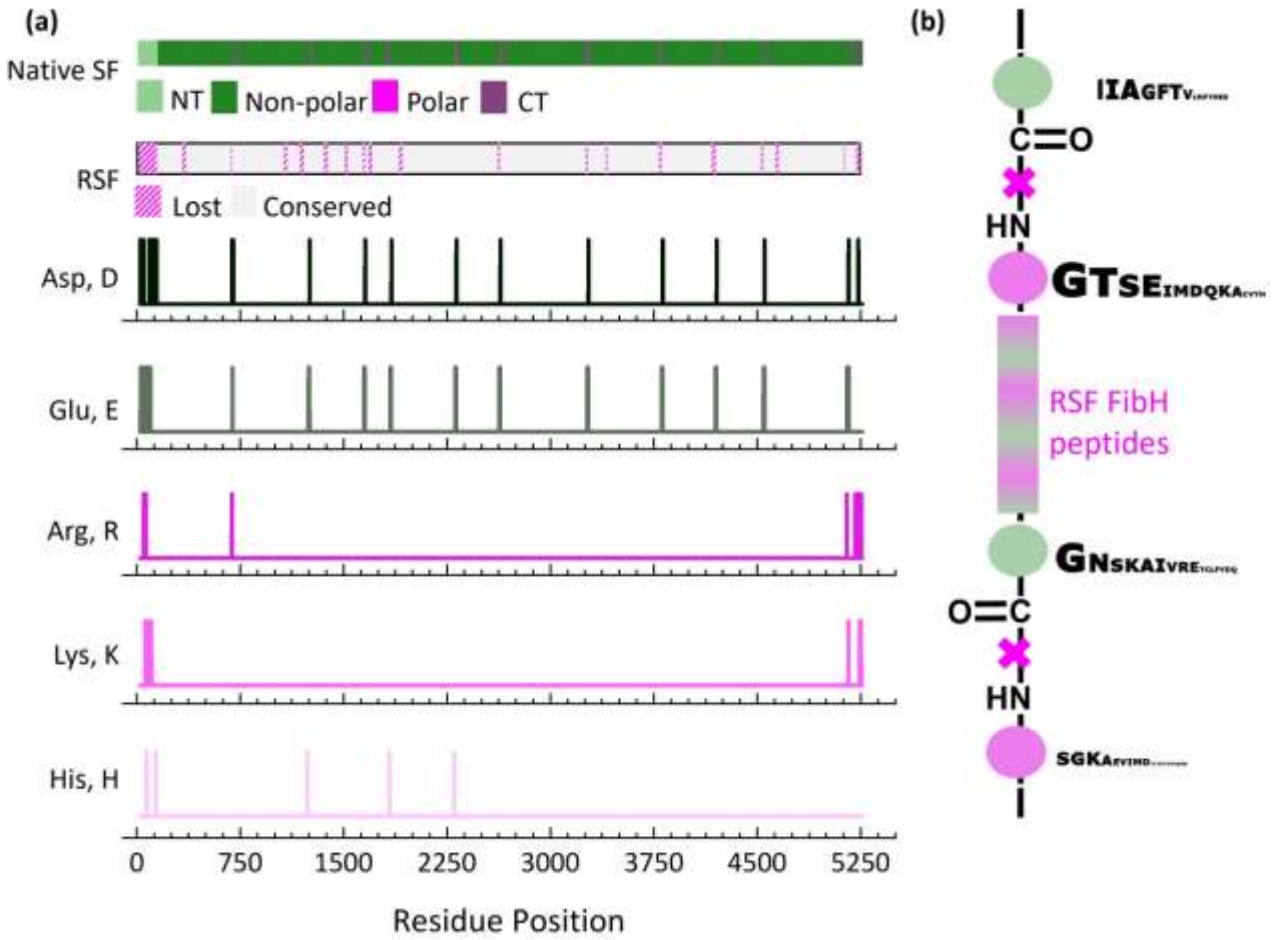

Figure S21.

Regeneration induced hydrolysis on fibroin. Carton showing the multidomain architecture of the primary structure of FibH (top), followed by an aligned cartoon representing the total sequence coverage from the hydrolysate peptides recovered from RSF. Below, different plots show several indicated residues' positions along the sequence of FibH (a). Scheme representing the recovered peptides from RSF, with logo representations of the observed residues at terminal positions of the detected peptides, and those expected from the retained chains (b)

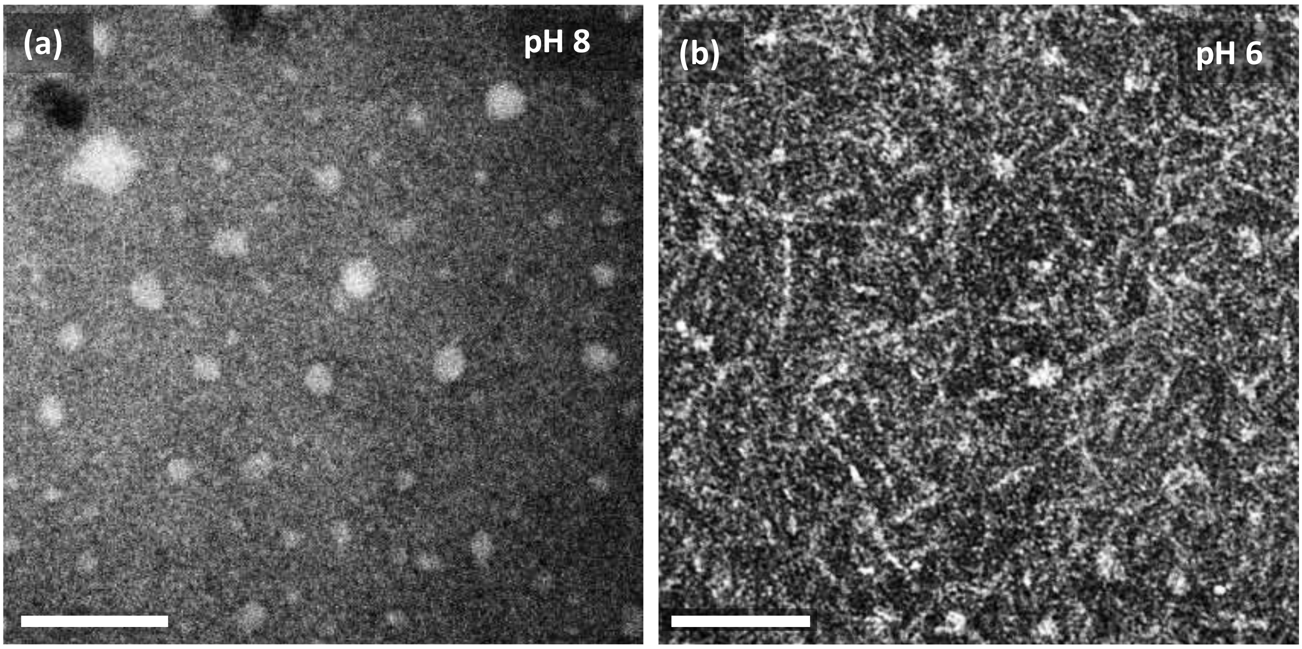

Figure S22.

TEM analysis of morphological changes in silk fibroin (NLSF). Negatively stained TEM images of globular units observed at pH 8 (a), and elongated fibrillar structures observed at pH 6 (b). Scale bars represent 100 nm in both cases.

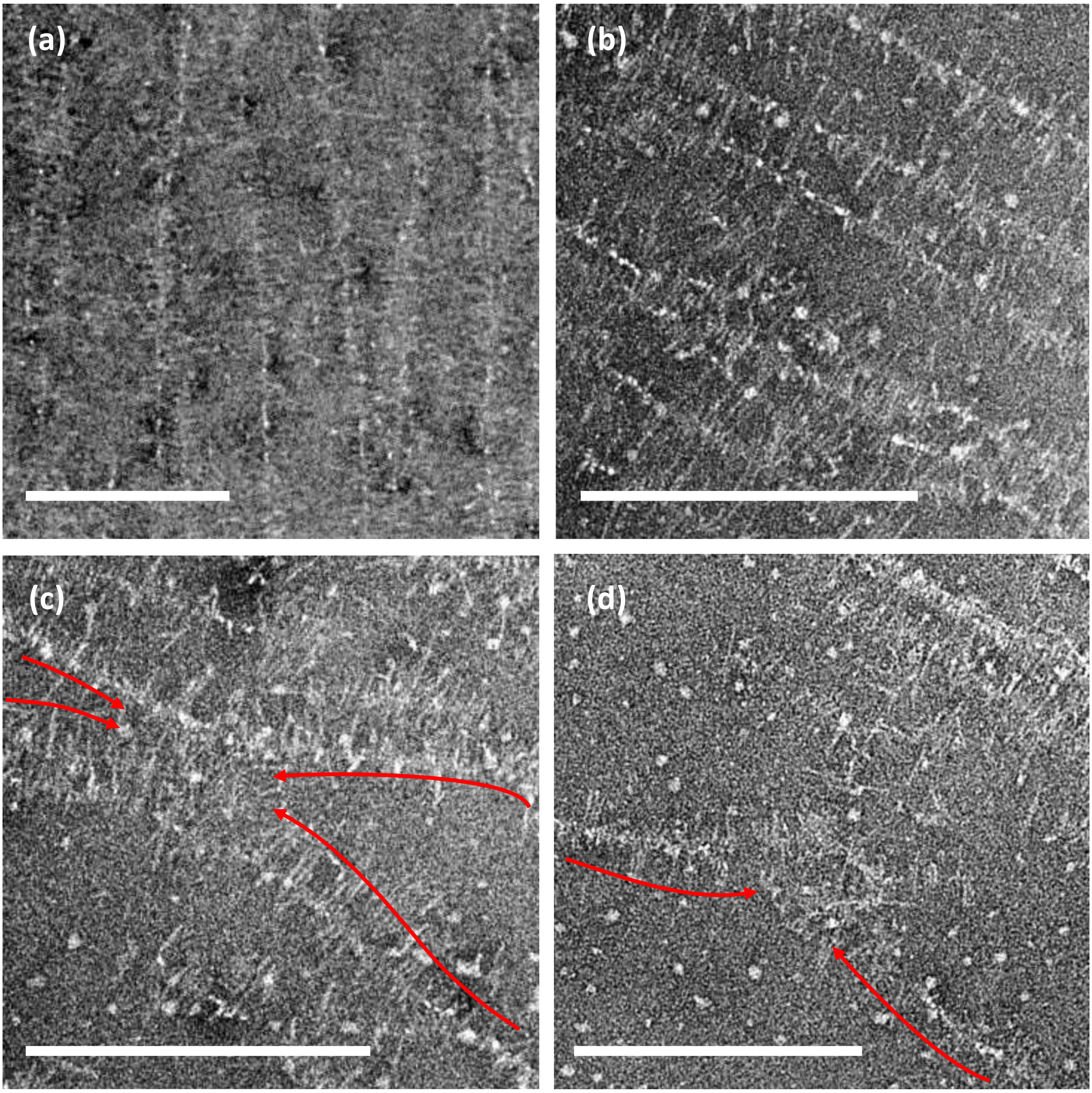

Figure S23.

TEM analysis of supramolecualr assemblies obbtained for NLSF. Negatively stained TEM images of intact supramolecular brush-like fibres (a, b) and illustrative images showing possible lateral interactions (c) and breakage of the structure (d), with arrows added as guides. Scale bars are 500 nm in (a-d).

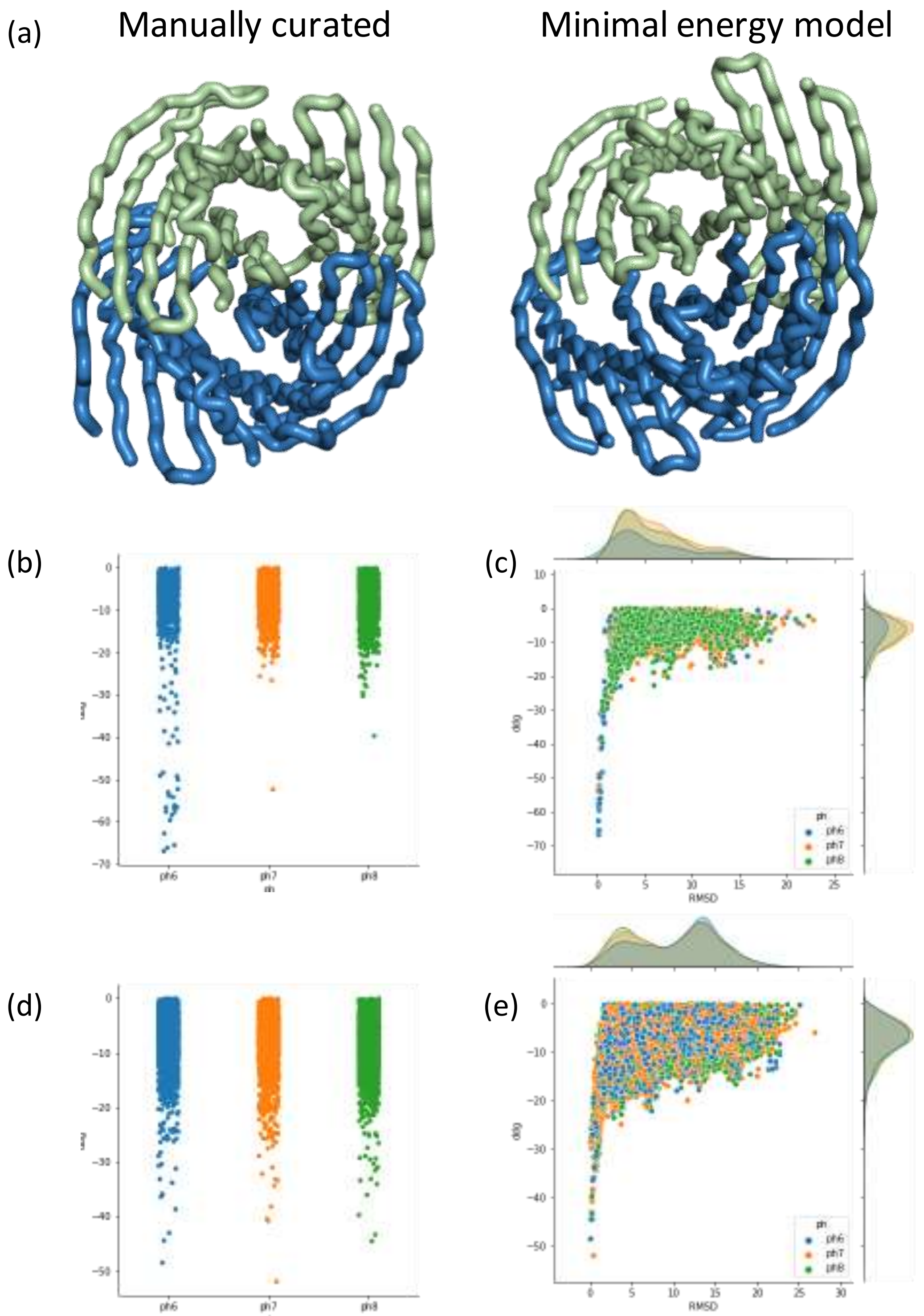

Figure S24.

N-terminal domain oligomerisation. (a) Ribbons representation of two stacked tetrameric units showing the manually curated model (left) and the equivalent relaxed and docked minimal energy model (right) as observed from the top (top). (b) Estimated rosseta binding free energies of docking simulations against pH for the n4/n4 interface. (c) Estimated rosetta binding free energies of docking simulations against residue median standard deviation (RMSD) of the different simulations with the manually curated and relaxed model as reference for the n4/n4 interface. (d) Estimated rosseta binding free energies of docking simulations against pH for the n2/n2 interface. (e) Estimated rosetta binding free energies of docking simulations against residue median standard deviation (RMSD) of the different simulations with the manually curated and relaxed model as reference for the n2/n2 interface.

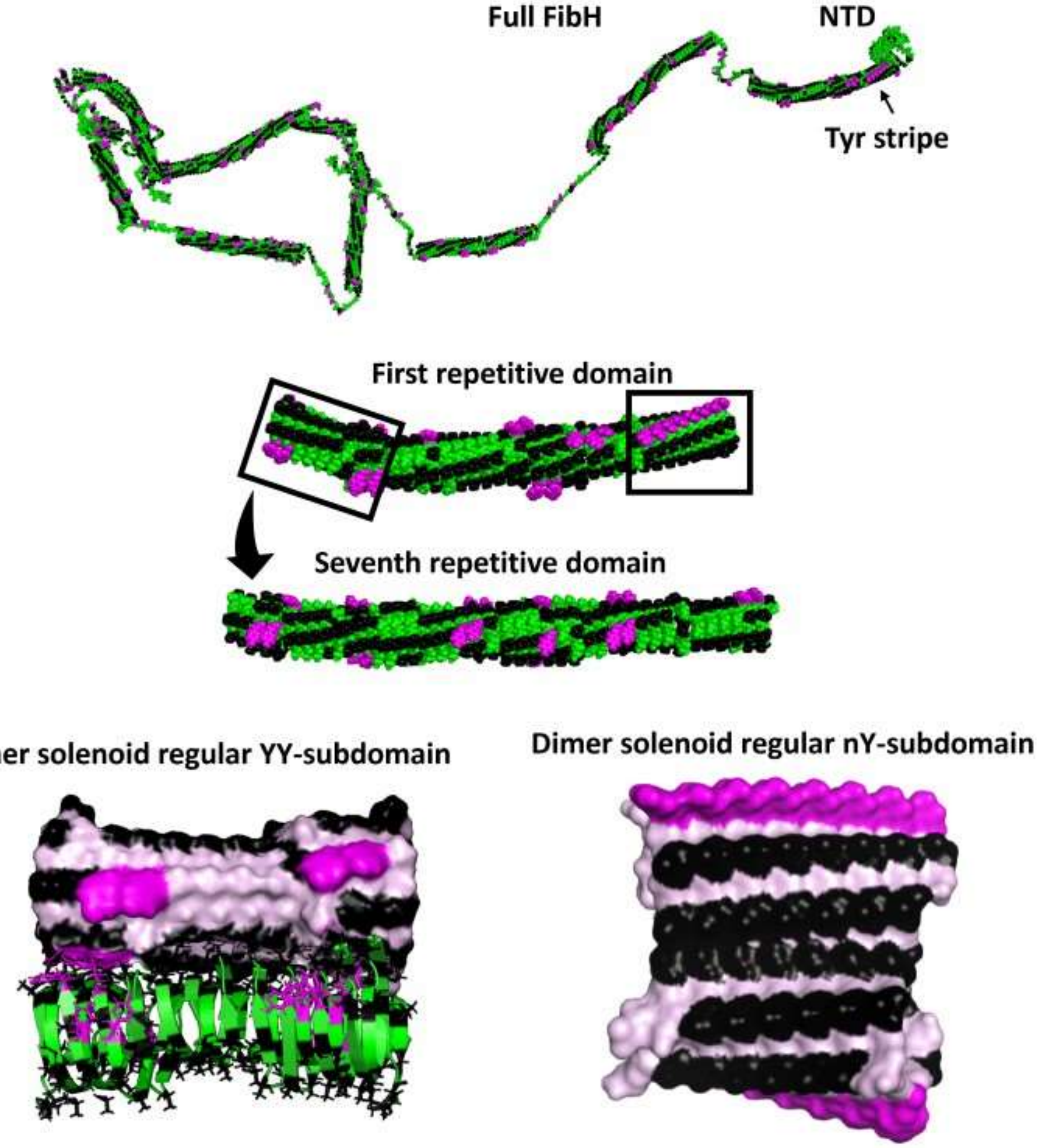

Figure S25.

The role of topology and Ala residues in driving lateral interactions. Complete proposed FibH surface model coloured to highlight Ala (black), Y (magenta) and Val (black) from all other residues (green or light pink) shown at the top. At the centre of the composite image, the detail of the first and sixth repetitive domain is shown, highlighting the detected subdomains and the difference in the spatial distribution of Y residues. At the bottom, two laterally docked models of the two different subdomains, showing binding at Ala interfaces with avoidance of Y residues; colour wheat or green represent all other residue but Ala and Y.

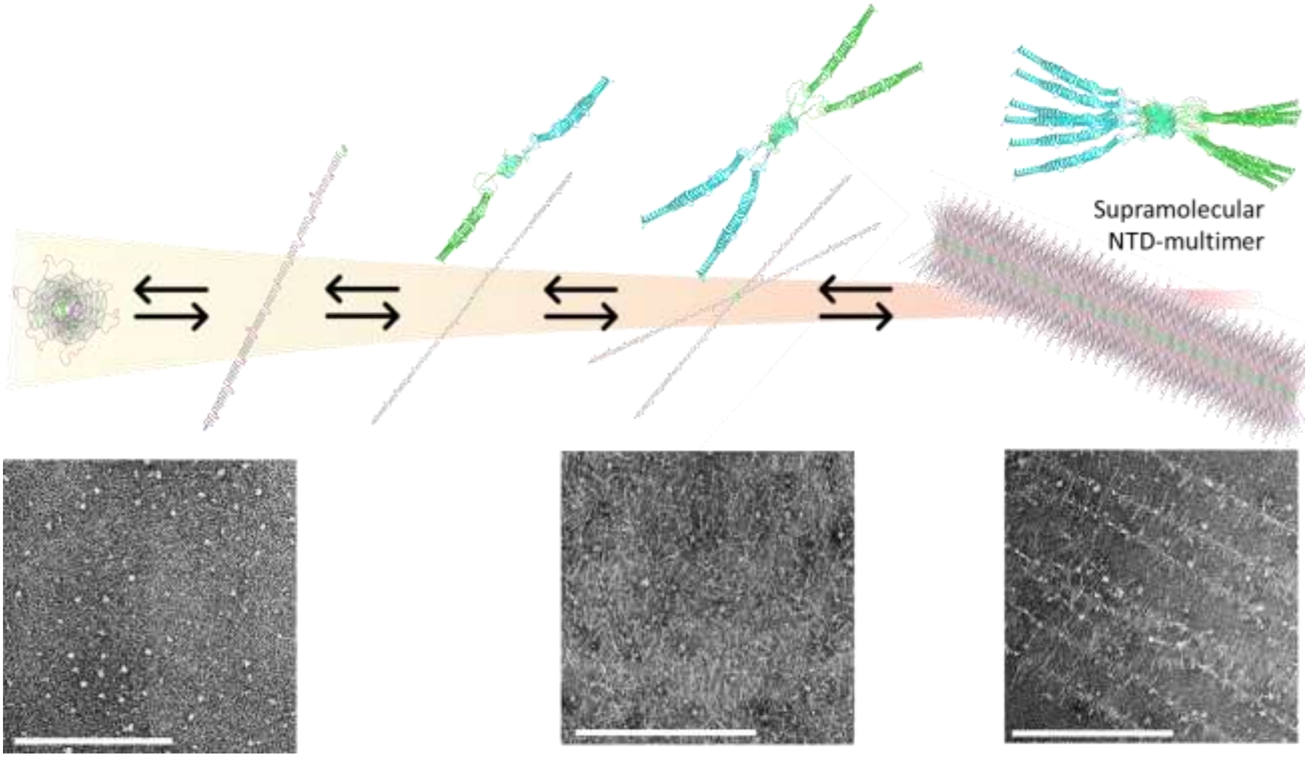

Figure S26.

Proposed NTD-driven self-assembly process: FibH, as a multidomain solenoidal molecule exists, takes a globular appearance with a diameter of about 20 nm at pH values above 7. At pH 7, the linkers are partially protonated, and the protein can extend and start participating in lower-order oligomer species. As pH continues to drop, assembly continues into the supramolecular bottlebrush fibres. PDB models represent only the NTD with the first repetitive domain, whereas drawings represent, schematically, the complete molecule. The background image's colour and shape allude to the pH gradient (from 8 to 6) and the anterior section of the silk gland (ASG), respectively. Negatively stained TEM images shown below are intended to show snapshots of the assembly process, and scale bars represent 500 nm.

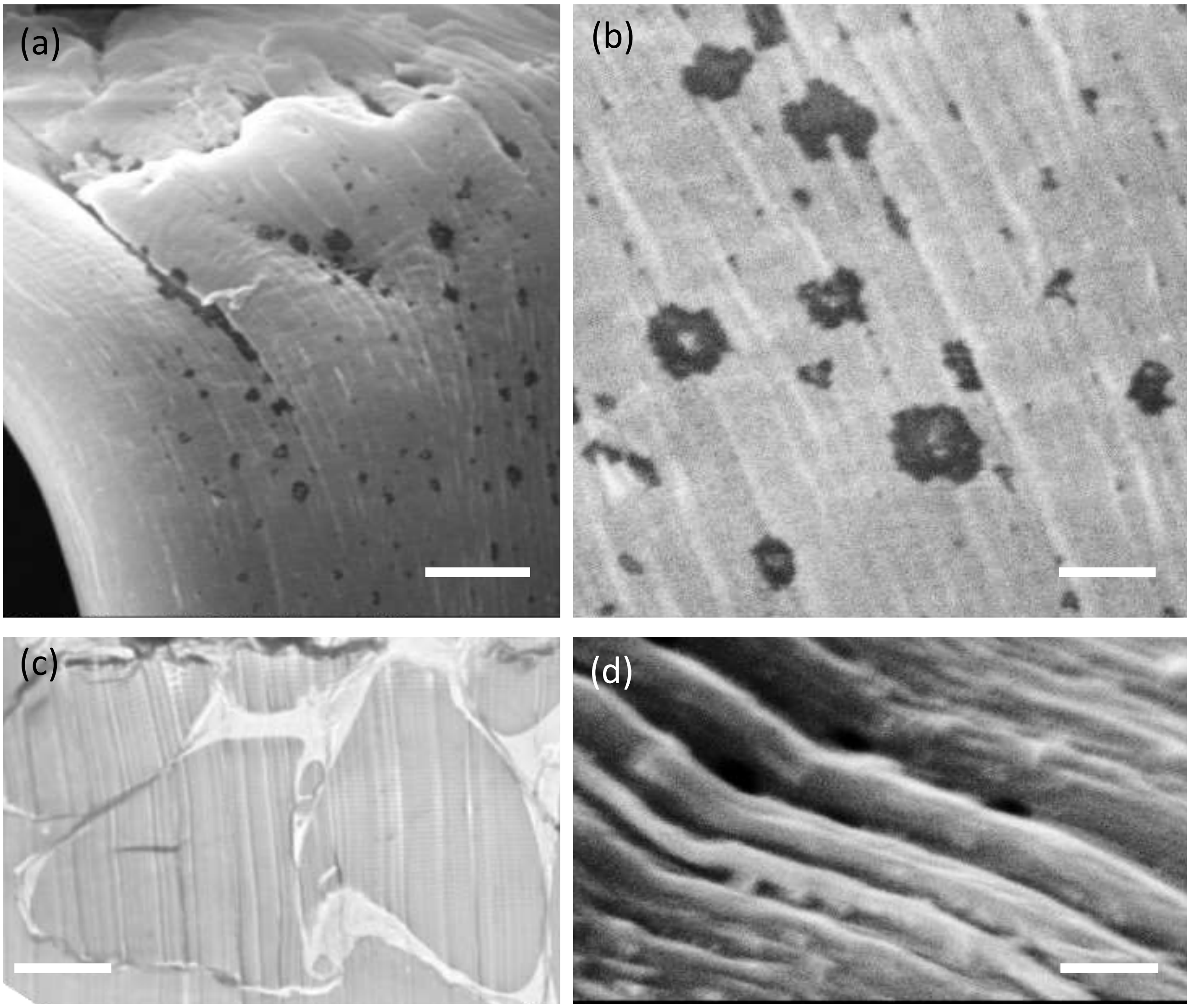

Figure S27.

Microscopy analysis of natural silk fibre. SED-SEM image of a sheared degummed fibre (a). SEM detail off the same fibre showing nanofibrillar texture and void formation during shearing (b). TEM image of a microtomed section of an embedded raw fibre showing twin fibroin fibres coated by a less electron-dense sericin layer (c). SEM image showing further detail of the nanofibrillar texture on a tilted specimen (D). Scale bars are 2 µm in (a) and 500 nm (b-d).

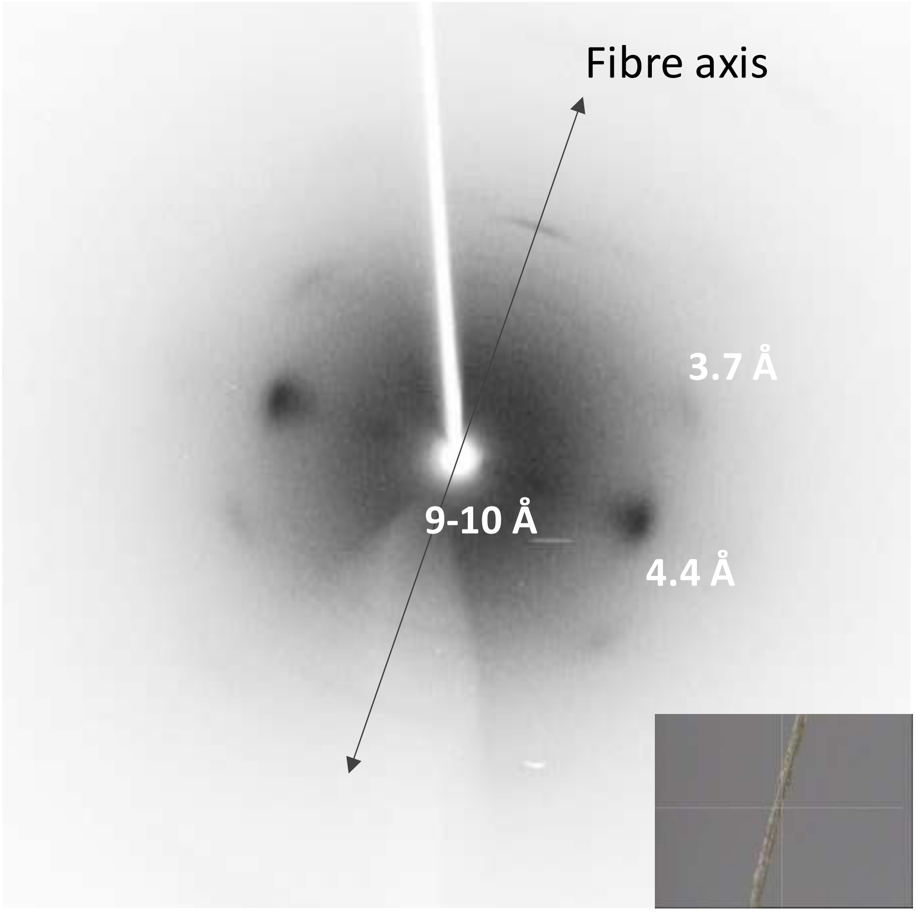

Figure S28.

Fibre X-ray diffraction of natural silk fibre. 2D X-ray diffraction pattern of a raw silk fibroin fibre with assigned distances.

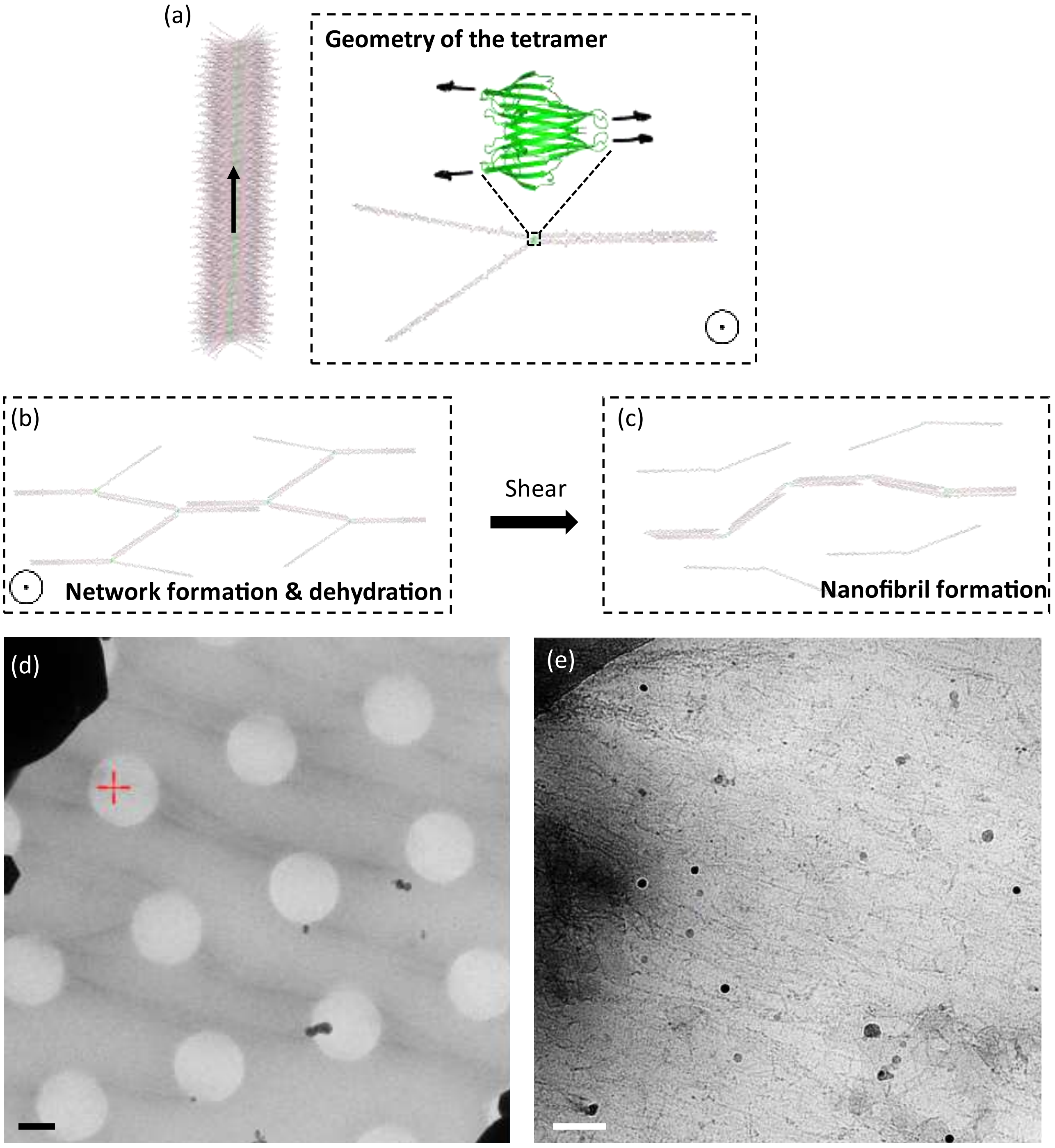

Figure S29.

Proposed network formation is driven by lateral interaction of solenoid units: Structure of a tetrameric unit within the supramolecular complex, showing the direction towards c-termini where the first repetitive domain would be located and the possible initiation intra-unit lateral interactions (a). Network of supramolecular fibres stabilized by lateral interactions of solenoids (b). Breakage of the NTD interactions and transformation to a flow-aligned solenoid network formation (c). Cryo-TEM image obtained from an apparent proto fibre at low magnification from NLSF (d), and at high magnification (e), showing what is believed to be evidence of the pre-extended solenoid network (NLSF) (e). Scale bar 1 µm in (d) and 100 nm in (e).

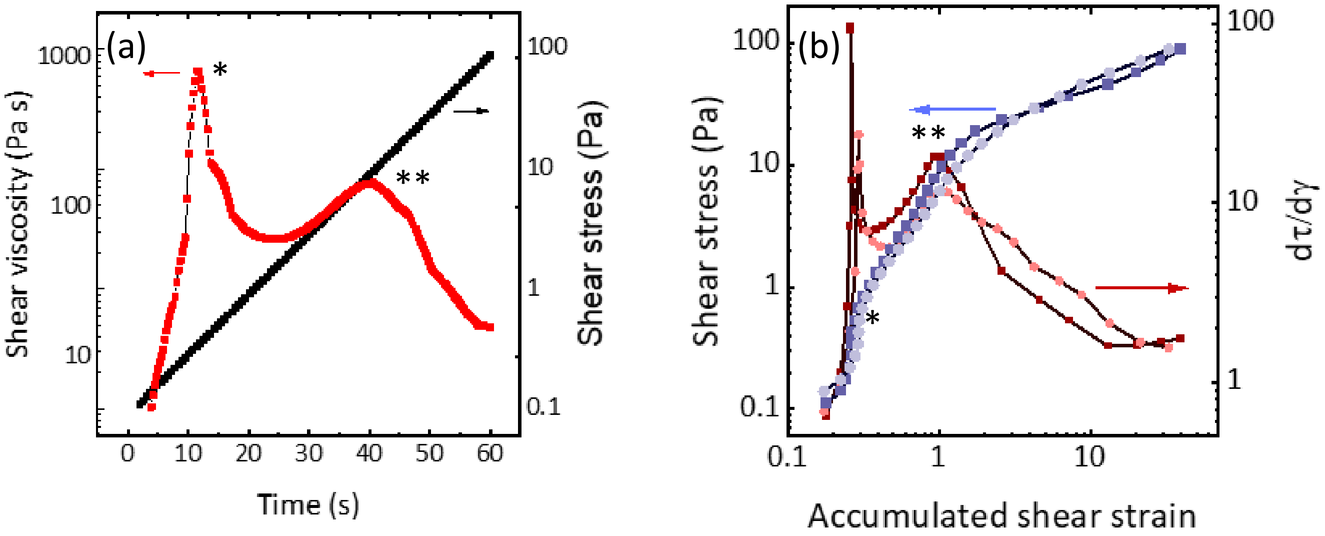

Figure S30.

Evidence of two yield stresses. Shear yield stress experiments conducted on a pH 7 viscoelastic sample: Shear viscosity and shear stress against time in a log-lineal scale, showing two yield points shown as maxima in viscosity (a). Shear stress and its derivative against accumulated shear strain for two independent samples showing a first sharp and a second broad yield (b).

Table S1. Different literature unit cell parameters for Silk I structures derived from X-ray diffraction.

Unit cell parameters								
a	b	c	α	β	γ	reference	Year	Molecular Feature
4.59	7.20	9.08	90	90	90	Konishi & Kurokawa[43]	1968	Helical folded strands
4.72	14.4	9.4	90	90	90	Lotz & Keith[30]	1971	Crankshaft model*
4.65	14.24	8.88	90	90	90	Okuyama et al.[31]	1988	Crankshaft-like*
4.79	4.79	22.2	90	90	120	Muñoz-Guerra et al.[44]	1988	Almost extended strand
8.94	6.46	11.26	90	90	90	Fossey et al.[45]	1991	Almost extended strand
22.66	5.70	20.82	90	90	90	He et al.[28]	1999	Almost extended strand

*Model used to derive the type-II β-turn model.

Table S2.

Reflections obtained from the oriented film diffraction pattern show typical Silk-I spacing.

Experimental results			
Equatorial		Meridional	
d-Spacings (Å)	Intensity	d-Spacings (Å)	Intensity
7.35	226	5.21	169
5.12	132	3.50	111
3.70	170	3.15	104
2.56	62	2.04	42
2.06	33		

Table S3.

NMR chemical shifts. Assigned chemical shifts for simplified motifs compared to those found in the literature [22].

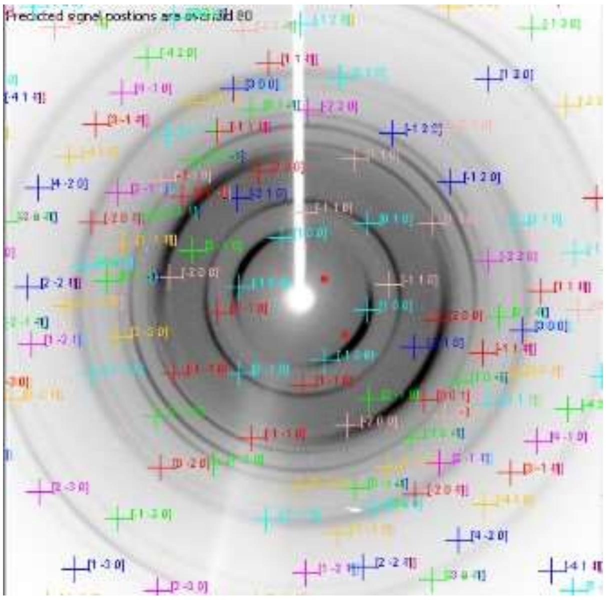

h	k	l	d-spacing																
**-5**	1	0	1.754	-2	0	-1	2.562	0	-2	1	2.064	2	-3	-1	1.721	4	-2	1	1.824
**-5**	2	0	1.788	-2	0	0	3.897	0	-1	-1	2.845	2	-3	0	1.996	4	-1	-1	1.863
**-4**	0	-1	1.691	-2	0	1	2.562	0	-1	0	5.196	2	-3	1	1.721	4	-1	0	2.227
**-4**	0	0	1.949	-2	1	-1	2.673	0	-1	1	2.845	2	-2	-1	2.226	4	-1	1	1.863
**-4**	0	1	1.691	-2	1	0	4.323	0	0	-2	1.7	2	-2	0	2.946	4	0	-1	1.691
**-4**	1	-1	1.863	-2	1	1	2.673	0	0	-1	3.4	2	-2	1	2.226	4	0	0	1.949
**-4**	1	0	2.227	-2	2	-1	2.226	0	0	1	3.4	2	-1	-1	2.673	4	0	1	1.691
**-4**	1	1	1.863	-2	2	0	2.946	0	0	2	1.7	2	-1	0	4.323	5	-2	0	1.788
**-4**	2	-1	1.824	-2	2	1	2.226	0	1	-1	2.845	2	0	1	2.562	5	-1	0	1.754
**-4**	2	0	2.162	-2	3	-1	1.721	0	1	0	5.196	2	1	-1	2.046				
**-4**	2	1	1.824	-2	3	0	1.996	0	1	1	2.845	2	1	0	2.563				
**-4**	3	0	1.824	-2	3	1	1.721	0	2	-1	2.064	2	1	1	2.046				
**-3**	-1	-1	1.701	-1	-2	-1	1.824	0	2	0	2.598	2	2	0	1.788				
**-3**	-1	0	1.964	-1	-2	0	2.162	0	2	1	2.064	3	-3	-1	1.701				
**-3**	-1	1	1.701	-1	-2	1	1.824	0	3	0	1.732	3	-3	0	1.964				
**-3**	0	-1	2.064	-1	-1	-1	2.464	1	-3	0	1.904	3	-3	1	1.701				
**-3**	0	0	2.598	-1	-1	0	3.576	1	-2	-1	2.226	3	-2	-1	2.064				
**-3**	0	1	2.064	-1	-1	1	2.464	1	-2	0	2.946	3	-2	0	2.598				
**-3**	1	-1	2.25	-1	0	-1	3.116	1	-2	1	2.226	3	-2	1	2.064				
**-3**	1	0	3	-1	0	0	7.794	1	-1	-1	2.945	3	-1	-1	2.25				
**-3**	1	1	2.25	-1	0	1	3.116	1	-1	0	5.892	3	-1	0	3				
**-3**	2	-1	2.064	-1	1	-1	2.945	1	-1	1	2.945	3	-1	1	2.25				
**-3**	2	0	2.598	-1	1	0	5.892	1	0	-1	3.116	3	0	-1	2.064				
**-3**	2	1	2.064	-1	1	1	2.945	1	0	0	7.794	3	0	0	2.598				
**-3**	3	-1	1.701	-1	2	-1	2.226	1	0	1	3.116	3	0	1	2.064				
**-3**	3	0	1.964	-1	2	0	2.946	1	1	-1	2.464	3	1	-1	1.701				
**-3**	3	1	1.701	-1	2	1	2.226	1	1	0	3.576	3	1	0	1.964				
**-2**	-2	0	1.788	-1	3	0	1.904	1	1	1	2.464	3	1	1	1.701				
**-2**	-1	-1	2.046	0	-3	0	1.732	1	2	-1	1.824	4	-3	0	1.824				
**-2**	-1	0	2.563	0	-2	-1	2.064	1	2	0	2.162	4	-2	-1	1.824				
**-2**	-1	1	2.046	0	-2	0	2.598	1	2	1	1.824	4	-2	0	2.162				

Table S4.

NMR chemical shifts. Assigned chemical shifts for simplified motifs compared to those found in the literature [22].

		Chemical shift (ppm, measured/literature*)													
		HN	HA	HB	HD	HE	HG	HD	HE	CA	CB	CG	CD	CE	N
GAGSGA	G1	8.4/8.46*	4.1/3.89*							44.8/44.7*					108.15/107.9*
	A2	8.25/8.25*	4.25/4.26*	1.3/1.33*						52.7/52.2*	19.1/18.6*				123.6/123.5*
	G3	8.48/8.49*	3.94/3.93*							44.8/44.7*					108.17/108.1*
	S4	8.3/8.3*	4.46/4.4*	3.86;3.91/3.81;3.87*						58.5/58*	63.9/63.4*				115.5/115.3*
	G5	8.54/8.55*	3.96/3.92*							44.8/44.7*					110.9/110.8*
	A6	8.25/8.25*	4.25/4.26*	1.3/1.33*						52.7/52.2*	19.1/18.6*				123.6/123.5*
GAGYGA	G1	8.41/8.36*	3.89/3.76;3.84*							44.8/44.7*					107.9/107.9*
	A2	8.18/8.17*	4.30/4.22*	1.35/1.3*						52.7/52.2*	19.0/18.6*				123.6/123.4*
	G3	8.41/8.39*	3.76/3.84							44.78/44.7*					108.14/107.9*
	Y4	8.1/8.1*	4.51/4.46*	2.9;3.04/2.85;2.98*	7.07/6.95*	6.8/6.71*		7.06	6.78	58.2/57.8*	38.7/38.3*		133.45	118.26	119.86/119.8*
	G5	8.41/8.41*	3.94/3.76;3.85*							44.8/44.7*					110.68/110.6*
	A6	8.18/8.18*	4.3/4.23*	1.3/1.3*						52.7/52.2*	19.1/18.6*				123.6/123.4*
GAGVGA	G1	8.47/8.39*	3.89/3.76;3.84*							44.8/44.7*					108.15/107.9*
	A2	8.19/8.18*	4.29/4.23*	1.3/1.3*						52.7/52.2*	19.1/18.6*				123.56/123.4*
	G3	8.47/8.37*	3.89/3.83*							44.8/44.7*					108.15/107.5*
	V4	7.98/7.97*	4.11/4.04*	2.06/2*			0.87/0.83*			62.36/62*	32.63/32.1*	20.36/20.1*			119.18/118.5*
	G5	8.52/8.51*	3.9/3.86*							44.8/44.7*					110.7/110.8*
	A6	8.19/8.18*	4.29/4.23*	1.3/1.3*						52.7/52.2*	19.1/18.6*				123.6/123.4*

Table S5.

Predicted chemical shifts for simplified motifs within the sixth repetitive domain. Chemical shifts were predicted using the webserver from SHIFTX2.

		HN	HA	HB	HD	HE	CA	CB	CD	CE	N
GAGSGA	G1	8.20	4.10				44.50				108.00
	A2	8.50	4.50	1.40			51.40	18.70			123.00
	G3	8.00	4.20				43.80				108.20
	S4	8.40	4.70	3.75/3.79			57.60	63.00			115.40
	G5	7.90	4.10				44.70				109.30
	A6	8.40	4.50	1.40			51.60	18.20			122.90
GAGYGA	G1	8.5	3.96				45.24				108.7
	A2	8.25	4.3	1.4			52.7	19.1			123.6
	G3	8.5	3.96				45.24				108.7
	Y4	8.1	4.5	2.91/3.03	7.06	6.78	58.16	38.77	133.45	118.26	119.88
	G5	8.5	3.96				45.24				108.7
	A6	8.25	4.3	1.4			52.7	19.1			123.6

Table S6.

Table with the logarithmic shifts used to calculate the master curves

Sol master curve			Gel master curve	
pH	Log(a_{pH}), Freq.	*Log(a_{pH}), G	*pH*	Log(a_{pH}), Freq.
7	0	1.83	7	0
8	-0.86	0	6.5	1.23
			6	3.14

*Given the suspected lower concentration of the sol fraction at this pH, and knowing that like viscosity, the moduli follow a power-law relationship with concentration, the moduli were scaled to match that of pH 8, however leaving the frequency domain unaffected.
